# Exploiting 1,2,3-Triazolium Ionic Liquids for Synthesis of Tryptanthrin and Chemoselective Extraction of Copper(II) Ions and Histidine-Containing Peptides

**DOI:** 10.3390/molecules21101355

**Published:** 2016-10-13

**Authors:** Hsin-Yi Li, Chien-Yuan Chen, Hui-Ting Cheng, Yen-Ho Chu

**Affiliations:** Department of Chemistry and Biochemistry, National Chung Cheng University, Chiayi 62102, Taiwan; lanbarla0708@gmail.com (H.-Y.L.); passercloud@gmail.com (C.-Y.C.); coconuthead222@hotmail.com (H.-T.C.)

**Keywords:** ionic liquid, Cu-free click reaction, tryptanthrin synthesis, affinity ionic liquid, copper ion, chemoselective extraction, peptide

## Abstract

Based on a common structural core of 4,5,6,7-tetrahydro[1,2,3]triazolo[1,5-*a*]pyridine, a number of bicyclic triazolium ionic liquids **1**–**3** were designed and successfully prepared. In our hands, this optimized synthesis of ionic liquids **1** and **2** requires no chromatographic separation. Also in this work, ionic liquids **1**, **2** were shown to be efficient ionic solvents for fast synthesis of tryptanthrin natural product. Furthermore, a new affinity ionic liquid **3** was tailor-synthesized and displayed its effectiveness in chemoselective extraction of both Cu(II) ions and, for the first time, histidine-containing peptides.

## 1. Introduction

**Figure 1:**
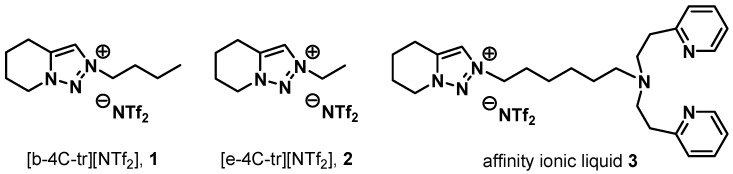
Structures of [b-4C-tr][NTf_2_] **1**, [e-4C-tr][NTf_2_] **2** and affinity ionic liquid **3**.

This paper describes the synthesis of bicyclic 1,2,3-triazolium ionic liquids **1**–**3** (Figure 1) [1], based on an intramolecular Hüisgen [3 + 2] cycloaddition (a Cu-free click reaction) [2,3], with specific aims to establish a common structural core for molecular diversity development of the ionic liquids and study their usefulness as reaction media for chemical synthesis and novel materials for biochemical applications. Ionic liquids are a class of polar solvents entirely made of ions, and many of them are liquids at room temperature [4,5]. Their ionic nature confers desirable properties to these liquids. They often possess wide liquid state ranges, high conductivity, excellent solubilization of many small molecules, good thermal stability, and negligible volatility [4,5]. The “green” character of ionic liquids largely refers to their very low vapor pressure, if any, and consequent inability to cause air pollution. Moreover, from the standpoint of organic synthesis, their blend of positive and negative charges makes these ionic liquids excellent microwave absorbers, giving rise to their recent emergence as superior alternatives to conventional molecular solvents for many microwave-assisted organic reactions [6].

In our research, we are interested in developing new ionic liquids or improving the synthesis of known ionic liquids and we have an ongoing program to evaluate ionic liquids as stable media for chemical synthesis [7,8,9,10,11] and as functional materials for biochemical applications [12,13]. We recently reported the preparation of bicyclic 1,2,3-triazolium ionic liquids in four steps with good yields [1]. Because of their intriguing chemical stability and our continuing interest in conducting research with this class of ionic liquids, we decided to develop a more generalized synthesis so that these bicyclic ionic liquids could be explored and studied systematically.

## 2. Results and Discussion

**Scheme 1:**
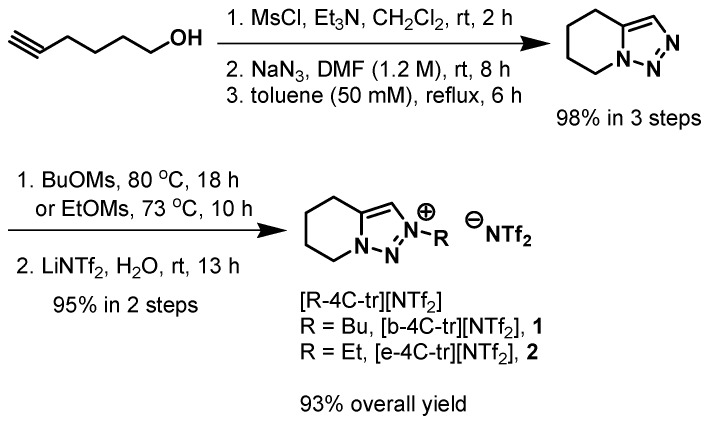
Synthesis of bicyclic 1,2,3-triazolium ionic liquids **1** and **2**.

Our synthesis of bicyclic 1,2,3-triazolium ionic liquids **1**, **2** is illustrated in Scheme 1. We aimed at demonstrating that these ionic liquids could be reliably prepared by a generalized synthetic route and therefore amended the previous synthesis by employing 5-hexyn-1-ol as the starting compound and successfully developed a 5-step synthesis of ionic liquids **1** and **2** in 93% overall isolated yield. First, the starting alcohol was mesylated using methanesulfonyl chloride in the presence of triethylamine at ambient temperature, followed by a two-step, one-pot reaction process involved in situ formation of 6-azidohex-1-yne using sodium azide and subsequently an intramolecular Cu-free, Hüisgen [3 + 2] cycloaddition in refluxing toluene for 6 h to afford the common 1,2,3-trazole core structure, 4,5,6,7-tetrahydro[1,2,3]triazolo[1,5-*a*]pyridine, with an excellent 98% isolated yield [14]. The progress of reactions could be readily monitored by TLC and, once reactions were complete, products were isolated and purified by straightforward extractions. The next reaction sequence proceeded with *N*-alkylation of the 1,2,3-triazole core by butyl methanesulfonate or ethyl methanesulfonate under heating conditions (80 °C for 18 h and 73 °C for 10 h, respectively) to cleanly produce the corresponding triazolium methanesulfonate salts, followed by metathesis with LiNTf_2_ in water at ambient temperature for 13 h to finally produce the desired ionic liquids **1** and **2** in excellent isolated yield (95%, in two steps). The overall isolated yield for this 5-step synthesis of both [b-4C-tr][NTf_2_] (**1**) and [e-4C-tr][NTf_2_] (**2**) ionic liquids was 93% (Scheme 1) [15]. Both ionic liquids obtained are liquids at room temperature. It is of note that this optimized synthesis of ionic liquids **1** and **2**, in our hands required no chromatographic separation.

We were interested in incorporating low-melting, hydrophobic [NTf_2_] anion in **1**, **2** primarily because of its water immiscibility, very low water content and low viscosity [4,5]. In addition, ionic liquids are known to be excellent microwave absorbers and thus should be useful for microwave-assisted organic synthesis. Accordingly, this high yielding preparation of ionic liquids allowed us to expediently investigate their applicability as ionic solvents for synthesis. In this work, we chose tryptanthrin as the target on which to investigate and support our design that synthesis of natural products could be promoted in ionic liquids [16].

Tryptanthrin is a biologically active quinazoline alkaloid isolated from a number of fungi (for instance *Schizophyllum commune*) and indigo plants (dried roots of *Polygonum tinctorium*, *Isatis indigotica* and *Strobilanthes cusia*) commonly known as “Ban Lan Gen” in Traditional Chinese Medicine [17,18]. In addition to its known antibacterial activity against various pathogenic microbes (e.g., *Helicobacter pylori*) and recently revealed antitubercular activity against *Mycobacterium tuberculosis* [19], tryptanthrin is a potent inhibitor of human cyclooxygenase 2 [20] and DNA topoisomerase I [21]. All these intriguing biological activities from tryptanthrin justify the study for its improved synthesis as a useful scaffold for drug discovery and the advanced development as anti-microbial, anti-inflammatory, and anti-neoplastic agents.

**Scheme 2:**
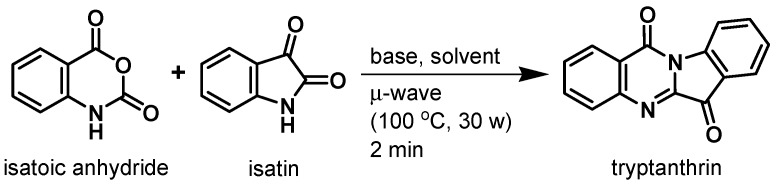
Microwave-assisted synthesis of tryptanthrin in ionic liquids **1** and **2**.

Many syntheses of tryptanthrin were reportedly achieved under basic and refluxing conditions (e.g., Et_3_N in refluxing toluene for 16 h) [19,22,23,24,25,26,27,28,29,30]. As one example, Srinivasan and co-workers reported the two steps synthesis of tryptanthrin in moderate (54%) isolated yield involving the use of LDA and starting from anthranilic acid, methyl anthranilate, and ethyl orthoformate [26]. Although tryptanthrin could also be prepared by following the established Eguchi aza-Wittig reaction protocol, its overall isolated yield however was low (32%) [31]. In the literature, the most common method for tryptanthrin synthesis is the reaction of isatoic anhydride with isatin in the presence of equimolar or excessive amounts of a base [17,18]. Scheme 2 illustrates our efficient microwaves-assisted synthesis of tryptanthrin carried out in ionic liquids **1** and **2**.

Table 1 summarizes our results obtained from the synthesis of tryptanthrin of which the middle two rings were concomitantly assembled from isatoic anhydride reaction with isatin in the presence or absence of base in both ionic (**1** and **2**) and common organic solvents (DMF and toluene). In this work, microwave irradiation was used to promote the double cyclization reactions and the polar, water-immiscible ionic liquids **1** or **2** were employed to deliberately drive the tryptanthrin-forming reaction to completion since water, once produced, was likely separated from the hydrophobic ionic liquid phase and rapidly evaporated under heating conditions. After several initial screenings of experimental conditions, it was quickly revealed that microwave irradiation (30 W) with the temperature controlled at 100 °C produced the best results.

It was found that with microwaves for a short 2 min, the reaction of isatoic anhydride with isatin and the base *N*,*N*-diisopropylethylamine (DIEA) in ionic liquid **1** cleanly furnished the desired natural product tryptanthrin in quantitative isolated yield after chromatography (entry 1, Table 1). An essentially identical yield (98%) of tryptanthrin synthesis was obtained when performed in ionic liquid **2** (entry 3, Table 1).

In our hands, the reaction with the inorganic base Cs_2_CO_3_ in ionic liquid **1** gave a lower product yield (81%, entry 2). When carried out in DMF, much lower isolated yields of tryptanthrin were obtained: 64% (with DIEA as base) and 21% (with Cs_2_CO_3_ as base), respectively (entries 4 and 5, Table 1). These results clearly demonstrated that ionic liquids **1** and **2** are excellent microwave absorbers, and these ionic solvents outperformed the molecular solvent DMF in the one-pot synthesis of tryptanthrin. Moreover, under our experimental conditions, toluene, a commonly used solvent for the synthesis of tryptanthrin, produced no trace of tryptanthrin (entries 8 and 9, Table 1) [19,22,23,24,25,26,27,28,29,30]. Although poor isolated yields of tryptanthrin were obtained in the ionic liquid **1** and DMF without the use of any base, ionic liquid **1** nevertheless produced a higher tryptanthrin yield than DMF: 19% versus 6%, respectively (entries 6 and 7, Table 1). This result obtained for the synthesis of tryptanthrin in ionic liquid **1** appears to be consistent with our recent report on the weak Lewis acidic nature of ionic liquids [32], which likely activates isatoic anhydride to facilitate tryptanthrin synthesis. Figure S1 (Supplementary Materials) illustrates a possible mechanism for the synthesis of tryptanthrin in ionic liquid without a base. Using thermogravimetric analysis (TGA), Figure S2 (Supplementary Materials) shows that both ionic liquids **1** and **2** have similar high temperature stability close to 400 °C (their thermal decomposition temperatures at 10% mass loss are 391 °C and 382 °C, respectively), which are far above the optimized microwave heating temperature for tryptanthrin synthesis (100 °C). These values are in good agreement with those reported for [NTf_2_]-based ionic liquids [33].

**Scheme 3:**
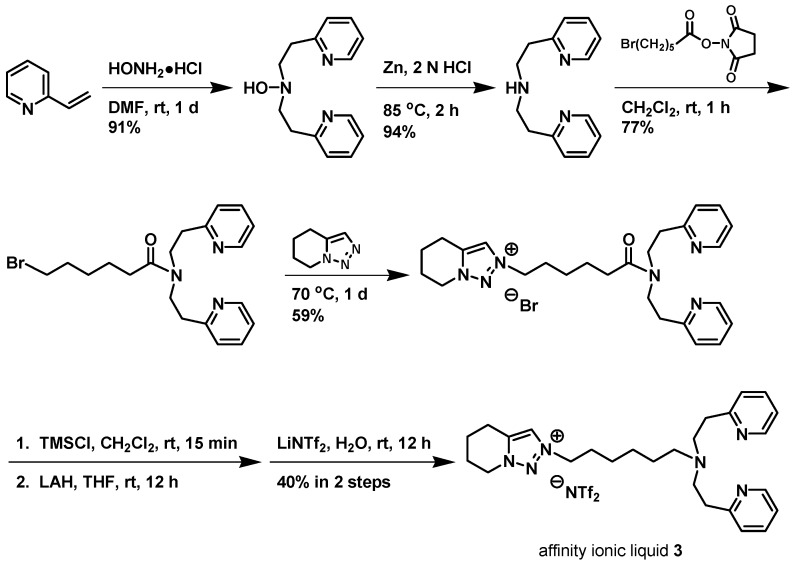
Synthesis of affinity ionic liquid **3**.

The success of our general approach to triazolium ionic liquid synthesis and its application for a one-pot synthesis of the natural product tryptanthrin prompted us further to initiate a preliminary study to evaluate its use as affinity ionic liquid for both metal ions and peptides. Here, we chose copper(II) as our initial target ion so that its chemoselective extraction based on molecular recognition could be facilitated using an affinity ionic liquid **3** (**AIL 3**). The copper ion is abundant in human serum as well as the brain [34], and plays an important role as an endogenous regulator of neuronal activity in living systems [35,36] and is closely correlated with the pathogenesis of Alzheimer’s disease [37]. Moreover, exposure to high copper concentrations can cause gastrointestinal disturbances, liver or kidney damage. Many fluorescence-based Cu(II) chemosensors have been reported and used for biological applications [38,39,40]. Scheme 3 illustrates our synthesis of affinity ionic liquid **3**.

Our synthesis of **AIL 3** began with the Michael addition of hydroxylammonium chloride to 2-vinylpyridine in DMF at room temperature for 1 d (91% yield), followed by a zinc reduction under acidic and heating conditions (94% yield) to afford the Cu(II)-recognition element, *N*,*N*-bis(2-(2-pyridyl)ethyl)amine, as a pale yellow oil (85% isolated yield in two steps) [41]. A straightforward amide bond coupling of the resulting bipyridylamine with *N*-succinimidyl 6-bromohexanoate was then carried out in dichloromethane at room temperature for 1 h (77% yield) and, after that, the direct alkylation with the bicyclotriazole developed in this work at 70 °C for 1 d (59% yield), a LAH reduction at room temperature for 12 h, and lastly an ion exchange in water at room temperature for 12 h (40% yield in 2 steps) to ultimately obtain **AIL 3**. In our hands, the overall isolated yield in the **AIL 3** synthesis was moderate: 16% over a total of six steps starting from 2-vinylpyridine. This **AIL 3** obtained is a viscous, pale yellow liquid at room temperature.

**Figure 2:**
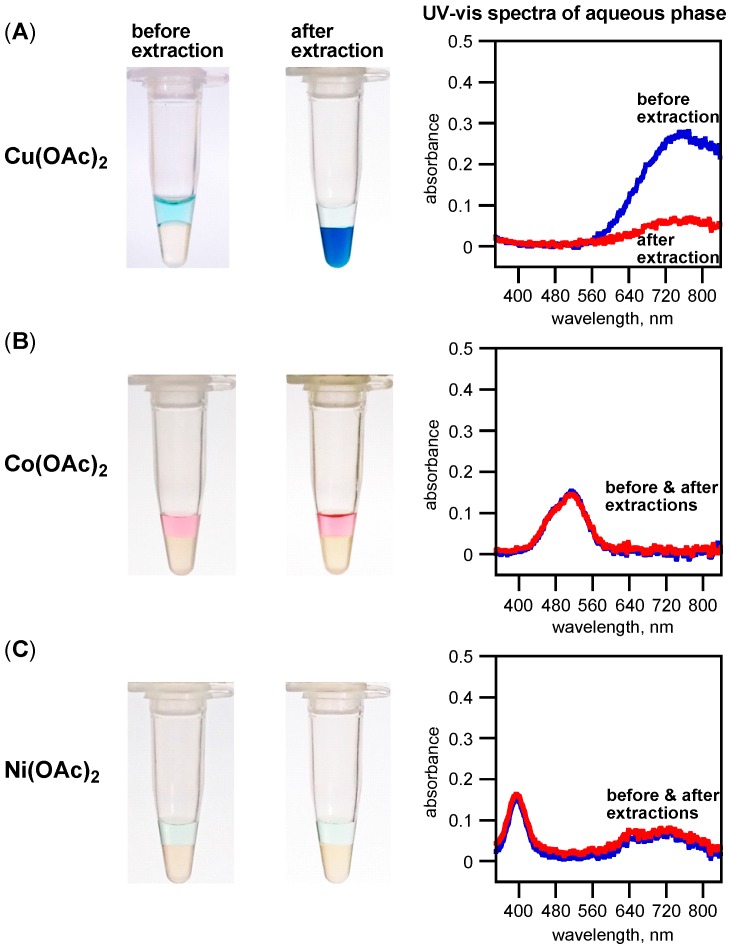
Optical photographic images and UV-vis spectra of chemoselective extractions of (**A**) Cu(OAc)_2_ (0.1 M); (**B**) Co(OAc)_2_ (0.25 M) and (**C**) Ni(OAc)_2_ (0.25 M) in H_2_O (25 µL each) by **AIL 3** (0.25 M) in ionic liquid **2** (25 µL), respectively. UV-vis spectra (1 mm path length) of aqueous layers were obtained by a NanoDrop 2000 spectrophotometer (Thermo Scientific, Waltham, MA, USA).

Results of both extraction photos and UV-vis spectra shown in Figure 2 unambiguously demonstrate that the **AIL 3** dissolved in [e-4C-tr][NTf_2_] ionic liquid **2** chemoselectively recognized Cu(II) ion in water (the upper layer) and, as a result, effectively extracted it into the ionic liquid (bottom layer). In these affinity extraction experiments, equal volumes of ionic liquid and water were first mixed, the resulting mixtures were then vigorously shaken and lastly centrifuged all at ambient temperature to accomplish the extraction. As shown in Figure 2B,C, Co(II) and Ni(II) ions remained unaffected in the upper water layer upon extraction, indicating that the partitioning of Co(II) and Ni(II) into ionic liquid phase containing **AIL 3** was negligible as demonstrated in their UV-vis spectra, and the recognition of **AIL 3** toward Cu(II) is clearly chemospecific (Figure 2A).

**Figure 3:**
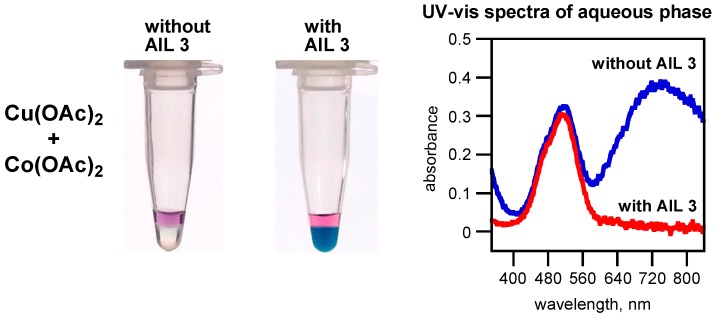
Optical photographic images and UV-vis spectra of affinity extraction of an aqueous solution (10 µL) containing Cu(OAc)_2_ (0.1 M) and Co(OAc)_2_ (0.5 M) by **AIL 3** (1.0 M) in ionic liquid **2** (10 µL).

Moreover, the affinity extraction experiment shown in Figure 3 further proved that, in the presence of both Cu(OAc)_2_ (0.1 M) and excessive Co(OAc)_2_ (0.5 M) in water, the complete transfer of only Cu(II) ion from the aqueous phase into the ionic liquid was entirely due to affinity recognition by **AIL 3** (1.0 M). This chemoselective extraction of Cu(II) ion and total retention of Co(II) were demonstrated in its UV-vis spectra and also apparent to the naked eyes (Figure 3).

**Scheme 4:**
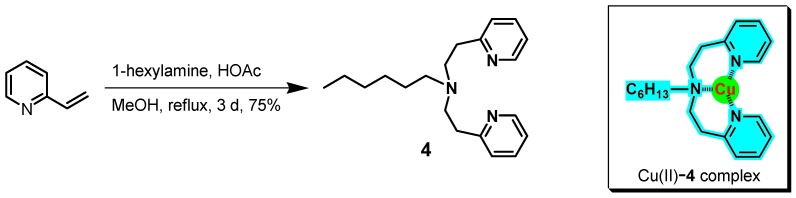
Synthesis of affinity ligand **4** and the proposed structure of Cu(II)-**4** complex.

To determine the stoichiometry of **AIL 3**-Cu(II) binding interaction, samples having a fixed concentration of an affinity ligand **4** (a surrogate of **AIL 3**) in DMSO/H_2_O (5:1, *v*/*v*) solution and various concentrations of Cu(OTf)_2_ were prepared to perform affinity extractions. As depicted in Scheme 4, affinity ligand **4** could be readily prepared with good 75% yield by a straightforward Michael addition of *n*-hexylamine to 2-vinylpyridine.

**Figure 4:**
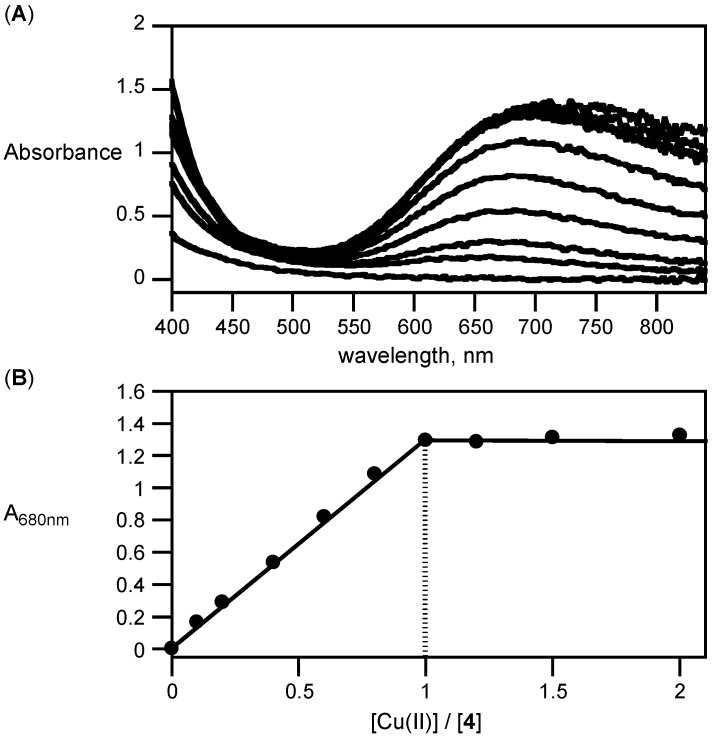
Measurement of the stoichiometry of affinity ligand **4** (100 mM) binding to Cu(OTf)_2_ (0, 10, 20, 40, 60, 80, 100, 120, 150, 200 mM) in DMSO/H_2_O (5:1, *v*/*v*) solution. (**A**) UV-vis spectra (1 mm path length) of samples with various (Cu(II))/(**4**) molar ratios were scanned, collected and overlapped by a NanoDrop 2000 spectrophotometer; (**B**) A plot of λ_max_ absorbance at 680 nm vs. (Cu(II))/(**4**) was used to determine the binding stoichiometry of **4** with Cu(OTf)_2_.

The results in Figure 4 show that as the ratio of (Cu(II))/(**4**) increased, the concentration (i.e., absorbance at 680 nm) of **4**-Cu(II) complex increased until the ratio reached its binding stoichiometry; beyond this value no more complex could be formed because all **4** has been fully titrated and associated with Cu(II) ion, and therefore excessive Cu(II) ion remained unbound in the solution (Figure 4A). This broad band having a λ_max_ at 680 nm with its growth in intensity was from the d-d transition of blue Cu(II) complex with affinity ligand **4** [38,39,40]. The plot of the A_680nm_ vs. the ratio of (Cu(II))/(**4**) revealed an abrupt change in the slope, corresponded to the binding stoichiometry of the system studied; that is, an expected 1:1 stoichiometry of this model **4**-Cu(II) binding interaction was obtained (Figure 4B). It is likely that the **4**-Cu(II) complex adopt a square-pyramidal coordination geometry in which affinity ligand **4** binds at the basal plane and two triflates occupy the remaining sites (Scheme 4) [41,42]. Overall, we were pleased that affinity ionic liquid **3** effectively dissolved and extracted Cu(II) into ionic liquid **2**, and its affinity ligand **4** tightly associated with Cu(II) to form a stable 1:1 complex.

**Figure 5:**
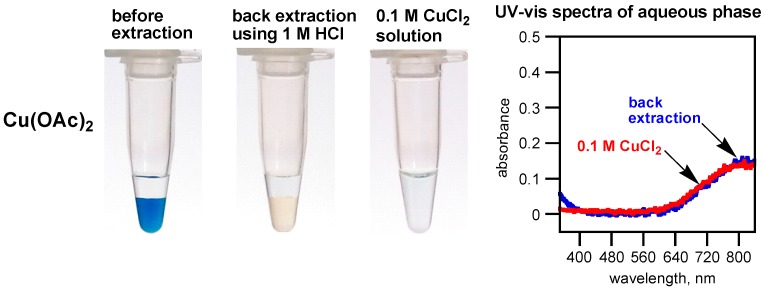
Optical photographic images and UV-vis spectra of competitive extraction of Cu(OAc)_2_ (0.1 M) and **AIL 3** (0.25 M) in ionic liquid **2** (25 µL) by 1 M HCl in H_2_O (25 µL). A photograph of 0.1 M CuCl_2_ aqueous solution (50 µL) was also included for comparison.

Figure 5 demonstrates that a complete recovery of Cu(II) ion from ionic liquid phase into the aqueous layer could be readily achieved by diminishing **AIL 3** binding with Cu(II) using a high concentration (1 M) of HCl. Thus, **AIL 3** in ionic liquid **2** could be readily regenerated.

**Figure 6:**
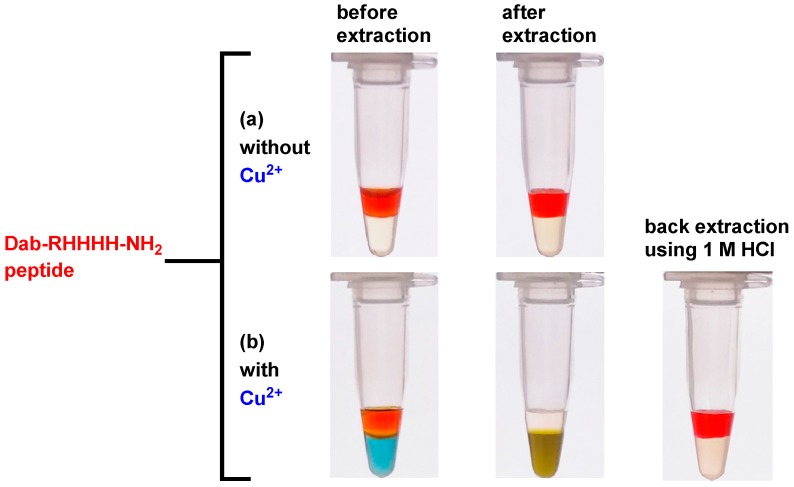
Affinity extraction of the dabsylated His-containing pentapeptide amide Dab-RHHHH-NH_2_ (1 mM) in 200 mM NaOAc buffer (pH 4.4) containing 10% DMSO (upper layer, 25 µL) by **AIL 3** (100 mM) solubilized in ionic liquid **1** (bottom layer, 25 µL). (**a**,**b**) are optical photographic images of peptide extraction into ionic liquid layer without and with chelated Cu^2+^ (50 mM), respectively. Back extraction of the peptide in ionic liquid layer into aqueous layer could be conveniently achieved using 1 M HCl solution (upper layer, 25 µL).

Since Cu(II)-iminodiacetic acid and related structural motifs are known for their abilities to bind to histidine residues in peptides and proteins [38,39,40,43], we envisaged that our **AIL 3**-Cu(II) complex system should be an effective platform for the one-step affinity isolation and purification of His-containing peptides by **AIL 3**. Preliminary results of affinity extraction of Cu(II)-chelated **AIL 3** with a dabsylated Dab-RHHHH-NH_2_ pentapeptide amide shown in Figure 6 clearly indicated that **AIL 3**-Cu(II) complex in ionic liquid **1** quantitatively extracted the peptide from aqueous phase into the bottom ionic liquid layer. Only with **AIL 3** in ionic liquid **1**, Dab-RHHHH-NH_2_ peptide remained totally unaffected in the upper water layer upon extraction. Furthermore, this peptide could be conveniently and completely recovered by 1 M HCl (Figure 6).

## 3. Materials and Methods

### 3.1. General Information

The ^1^H-NMR and ^13^C-NMR spectra were recorded at 400 MHz and 100 MHz, respectively on an Avance DPX 400 spectrometer (Bruker, Billerica, MA, USA) in deuterated solvents (CDCl_3_ or DMSO-*d*_6_) using TMS as the internal standard. The chemical shift (δ) for ^1^H and ^13^C are given in ppm relative to residual signal of the solvent. Coupling constants are given in Hz. The following abbreviations are used to indicate the multiplicity: s, singlet; d, doublet; t, triplet; q, quartet; m, multiplet; dd, doublet of doublets; td, triplet of doublets; dt, doublet of triplets; ddd, doublet of doublet of doublets; brs, broad signal. Microwave irradiation experiments were performed using a CEM Discover microwave reactor from CEM Corporation (Matthews, NC, USA). The reactions were monitored using TLC silica gel 60 F254 (Merck, Darmstadt, Germany). Evaporation of solvents was performed under reduced pressure. Melting points were measured and recorded by the OptiMelt MPA-100 apparatus (Standford Research Systems, Sunnyvale, CA, USA) and uncorrected. All starting materials, reagents and solvents were purchased from commercial sources and used as such without further purification. Unless otherwise stated, all reactions were carried out under inert atmosphere using anhydrous solvents.

### 3.2. Synthesis of 4,5,6,7-Tetrahydro[1,2,3]triazolo[1,5-a]pyridine

To a ice chilled solution of 5-hexyn-1-ol (4.77 g, 48.57 mmol) and triethylamine (13.6 mL, 97.14 mmol) in dichloromethane (80 mL) was slowly added solution of methanesulfonyl chloride (6.68 g, 58.28 mmol) in dichloromethane (40 mL). The resulting solution was aged first for 30 min in ice bath and then brought to ambient temperature for another 2 h. The reaction solution was washed sequentially with citric acid (10 wt %, 3 × 15 mL), sodium bicarbonate (10 wt %, 3 × 15 mL), then dried over Na_2_SO_4_. After filtration, the solvent was removed under reduced pressure to afford high purity product (8.47 g, quantitative yield) with no need for further purification.

The obtained sulfonate (8.47 g, 48.05 mmol) was dissolved in DMF (40 mL) and then sodium azide (6.25 g, 96.10 mmol) was added. The substitution reaction was carried out at ambient temperature for 8 h. The resulting solution was then diluted with toluene (962 mL) and refluxed for 6 h. After completion of reaction, solvents were removed in vacuo. Dichloromethane (100 mL) was added and the resulting solution was washed with water (4 × 20 mL). Organic layers was dried over Na_2_SO_4_, filtered and concentrated under reduced pressure to afford high purity product 4,5,6,7-tetrahydro-[1,2,3]triazolo[1,5-*a*]pyridine as a light brown liquid (5.77 g, 98% yield) with no need for further purification. ^1^H-NMR (CDCl_3_) δ 1.86–1.97 (m, 2H, CH_2_), 2.02–2.14 (m, 2H, CH_2_), 2.84 (t, *J* = 6.4 Hz, 2H, CH_2_C=C), 4.37 (t, *J* = 6.2 Hz, 2H, NCH_2_), 7.44 (s, 1H, aryl H); ^13^C-NMR (DMSO-*d*_6_) δ 19.80, 19.82, 22.4, 45.7, 130.3, 133.0.

### 3.3. Synthesis of Ionic Liquid [b-4C-tr][NTf_2_] *(**1**)*

Solution of methanesulfonyl chloride (5.23 g, 45.66 mmol) in dichloromethane (55 mL) was slowly added to the ice bathed solution of butanol (2.82 g, 38.05 mmol) and triethylamine (10.6 mL, 76.10 mmol) in dichloromethane (55 mL). The resulting solution was allowed to react in an ice bath for 30 min and then brought to ambient temperature for another 2 h. Sequentially, the reaction solution was washed with citric acid (10 wt %, 3 × 15 mL), sodium bicarbonate (10 wt %, 3 × 15 mL), then dried over Na_2_SO_4_. After filtration, the solvent was removed under reduced pressure to afford high purity product (5.77 g, quantitative yield) with no need for further purification. To a round bottle flask containing 4,5,6,7-tetra-hydro[1,2,3]triazolo[1,5-*a*]pyridine (4.13 g, 33.52 mmol) was added butyl methanesulfonate (5.73 g, 37.64 mmol). The alkylation reaction was carried out at 80 °C for 18 h. After completion of reaction, the mixture was allowed to cool and then ether was added to extract and wash away remaining starting reagents, if any. Ether layer (140 mL in total) was removed and then the ionic liquid was concentrated under reduced pressure to afford the desired [b-4C-tr][OMs] (8.94 g, 97% yield). To the solution of [b-4C-tr][OMs] (8.94 g, 32.45 mmol) in water (5 mL) was added LiNTf_2_ (10.59 g, 36.87 mmol). The metathesis reaction was allowed to proceed at ambient temperature for 13 h. After completion of the ion exchange, the solution mixture was extracted with dichloromethane (3 × 5 mL). The combined organic layers were dried over Na_2_SO_4_. Evaporation of the solvent in vacuo afford the ionic liquid **1**, [b-4C-tr][NTf_2_] (14.54 g, 98% yield). Light brown liquid; ^1^H-NMR (DMSO*-d*_6_) δ 0.91 (t, *J* = 7.4 Hz, 3H, CH_2_C*H*_3_), 1.25–1.37 (m, 2H, C*H*_2_CH_3_), 1.81–1.93 (m, 4H, 2 × CH_2_), 2.03–2.14 (m, 2H, CH_2_), 2.94 (t, *J* = 6.4 Hz, 2H, CH_2_C=C), 4.51 (t, *J* = 6.1 Hz, 2H, NCH_2_), 4.58 (t, *J* = 7.1 Hz, 2H, NCH_2_), 8.68 (s, 1H, aryl H); ^13^C-NMR (CDCl_3_) δ 13.0, 17.8, 19.2, 19.8, 21.2, 30.8, 48.9, 53.6, 119.7 (q, *J*_CF_ = 319 Hz, CF_3_), 127.1, 140.4; FAB-HRMS *m*/*z* [M]^+^ calcd for C_10_H_18_N_3_ 180.1495, found 180.1501.

### 3.4. Synthesis of Ionic Liquid [e-4C-tr][NTf_2_] *(**2**)*

Methanesulfonyl chloride (2.59 g, 22.62 mmol) in dichloromethane (25 mL) was slowly added to the ice bathed solution of ethanol (0.87 g, 18.85 mmol) and triethylamine (5.3 mL, 37.70 mmol) in dichloromethane (20 mL). The resulting solution was allowed to react in ice bath for 30 min and then brought to ambient temperature for another 2 h. Sequentially, the reaction solution was washed with citric acid (10 wt %, 3 × 15 mL), sodium bicarbonate (10 wt %, 3 × 15 mL), then dried over Na_2_SO_4_. After filtration, the solvent was removed under reduced pressure to afford high purity product (2.03 g, 87% yield) without further purification. To a round bottle flask containing 4,5,6,7-tetra-hydro[1,2,3]triazolo[1,5-*a*]pyridine (1.75 g, 14.23 mmol) was added ethyl methanesulfonate (2.01 g, 16.2 mmol, 1.14 equiv.). The alkylation reaction was performed at 73 °C for 10 h. After completion of reaction, the reaction mixture was allowed to cool and then ether was added to extract and wash away unreacted starting reagents, if any. Organic layer (80 mL in total) was then removed and the ionic liquid phase was concentrated under reduced pressure to afford [e-4C-tr][OMs] (3.43 g, 98% yield). To the solution of [e-4C-tr][OMs] (3.43 g, 13.88 mmol) in water (2 mL) was added LiNTf_2_ (4.38 g, 15.27 mmol). The metathesis reaction was allowed to proceed at ambient temperature for 13 h. After completion of the ion exchange, the solution mixture was extracted with dichloromethane. The combined organic layers were dried over Na_2_SO_4_. Evaporation of the solvent in vacuo afford the desired ionic liquid **2**, [e-4C-tr][NTf_2_] (5.79 g, 97% yield). Light brown liquid; ^1^H-NMR (CDCl_3_) δ 1.65 (t, *J* = 7.5 Hz, 3H, CH_3_), 1.99–2.09 (m, 2H, CH_2_), 2.18–2.29 (m, 2H, CH_2_), 3.03 (t, *J* = 6.4 Hz, 2H, CH_2_C=C), 4.51 (t, *J* = 6.2 Hz, 2H, NCH_2_), 4.57 (q, *J* = 7.5 Hz, 2H, C*H*_2_CH_3_), 8.19 (s, 1H, aryl H); ^13^C-NMR (CDCl_3_) δ 13.9, 17.8, 19.8, 21.1, 48.9, 49.2, 119.6 (q, *J*_CF_ = 319 Hz, CF_3_), 126.7, 140.4; EI-HRMS *m*/*z* [M]^+^ calcd for C_8_H_14_N_3_ 152.1188, found 152.1183.

### 3.5. One-Pot Synthesis of Tryptanthrin in [b-4C-tr][NTf_2_] Ionic Liquid *(**1**)*

To a microwave reaction vessel with a magnetic stirring bar was added isatin (14.80 mg, 0.1006 mmol), isatoic anhydride (17.06 mg, 0.1046 mmol), DIEA (13.00 mg, 0.1006 mmol) and [b-4C-tr][NTf_2_] **1** (50 µL, 2.01 M). The vessel was placed inside a CEM Discover single-mode microwave synthesizer equipped with a magnetic stirrer, where it was exposed to microwaves at 100 °C (30 watts) for 2 min. The reaction mixture was cooled and then purified by flash column chromatography (ethyl acetate/hexane = 1:5) to obtain tryptanthrin (24.9 mg, quantitative yield) as yellow crystalline solid. Yellow crystalline solid; mp 266–268 °C; ^1^H-NMR (CDCl_3_) δ 7.43 (t, *J* = 7.8 Hz, 1H, aryl H), 7.67 (td, *J* = 7.7, 1.4 Hz, 1H, aryl H), 7.78 (td, *J* = 7.9, 1.4 Hz, 1H, aryl H), 7.85 (td, *J* = 7.7, 1.4 Hz, 1H, aryl H), 7.91 (dd, *J* = 7.0, 1.4 Hz, 1H, aryl H), 7.91 (dd, *J* = 7.0, 1.4 Hz, 1H, aryl H), 8.03 (dd, *J* = 8.0, 1.4 Hz, 1H, aryl H), 8.43 (dd, *J* = 8.0, 1.4 Hz, 1H, aryl H), 8.62 (d, *J* = 8.1 Hz, 1H, aryl H); ^13^C-NMR (CDCl_3_) δ 117.9, 121.9, 123.7, 125.4, 127.2, 127.5, 130.2, 130.7, 135.1, 138.2, 144.3, 146.3, 146.6, 158.0, 182.5; EI-HRMS *m*/*z* [M + H]^+^ calcd for C_15_H_8_N_2_O_2_ 248.0586, found 248.0586.

### 3.6. Synthesis of Affinity Ionic Liquid ***3***

To a solution of hydroxylamine hydrochloride (1.0 g, 14.4 mmol) in DMF (2 mL) was added 2-vinylpyridine (2 equiv.). The resulting solution was allowed to react at ambient temperature for 24 h. After the reaction was completed, the reaction mixture was neutralized with sodium bicarbonate (10 wt %, 20 mL). The product was extracted from the mixture with dichloromethane, and the resulting organic phase was dried over Na_2_SO_4_ and evaporated to dryness. Recrystallization of the residue from CH_2_Cl_2_/hexanes yielded *N*,*N*-bis(2-(pyridin-2-yl)ethyl)hydroxylamine (3.10 g, 91% yield) as pale yellow solid. A mixture of *N*,*N*-bis(2-(pyridin-2-yl)ethyl)hydroxylamine (1.2 g, 4.93 mmol) and zinc powder (1.47 g, 22.4 mol) in 2 N HCl (10 mL) was stirred at 85 °C for 2 h. After cooling, the solution was adjusted to pH 10 using concentrated aqueous ammonia, and the resulting mixture extracted with CH_2_Cl_2_. The organic washings were dried over Na_2_SO_4_ and the solvent was removed under reduced pressure to afford *N*,*N*-bis(2-(pyridin-2-yl)ethyl)amine (1.07 g, 94% yield) as a yellow oil. To a solution of 6-bromohexanoic acid *N*-hydroxysuccinimide ester (0.72 g, 2.48 mmol) in dichloromethane was added *N*,*N*-bis(2-(pyridin-2-yl)ethyl)amine (2.72 mmol). The resulting solution was allowed to react at ambient temperature for 1 h, then washed with sodium bicarbonate (10 wt %), brine and finally dried over Na_2_SO_4_. After filtration and solvent evaporation under reduced pressure, the crude product mixture was purified by flash column chromatography using ethyl acetate as the eluent to afford the desired amide product (0.432 g, 77% yield) as a pale yellow oil. To a round bottle containing 4,5,6,7-tetrahydro-[1,2,3]triazolo[1,5-*a*]pyridine (0.77 g, 6.26 mmol) was added the above obtained amide product (0.72 g, 1.78 mmol). The mixture was stirred at 70 °C for 24 h. The resulting slurry was washed with ether and purified by column chromatography using methanol/dichloromethane (1:20, *v*/*v*) as the solvent to afford the desired ionic salt (0.56 g, 59% yield). To a solution of the ionic salt (559 mg, 1.06 mmol) in dry CH_2_Cl_2_ (2 mL) was added TMSCl (138 mg, 1.27 mmol) in ice bath. After 15 min, 1 M LAH in THF (5 equiv.) was added slowly to the mixture with syringe. The resulting solution was allowed to react at ambient temperature for 12 h. After 12 h, the solvent was removed under reduced pressure to afford solid mixture. Deionized water was added to the resulting mixture for quenching LAH in ice bath. After filtration, LiNTf_2_ (331.5 mg, 1.16 mmol) was added to filtrate. The reaction was allowed to proceed the ion exchange at ambient temperature for 12 h. After completion of ion exchange, the mixture was extracted with CH_2_Cl_2_, the organic phases were combined and dried with Na_2_SO_4_. After filtration and solution concentration, the mixture purified using silica column chromatography using methanol/dichloromethane (1:10, *v*/*v*) as the solvent to give the targeted affinity ionic liquid **3** (0.28 g, 40% yield in two steps) as a yellow oil. ^1^H-NMR (CDCl_3_) δ 1.39–1.55 (m, 4H, CH_2_), 1.80–1.93 (m, 2H, CH_2_), 1.97–2.10 (m, 4H, CH_2_), 2.17–2.27 (m, 2H, CH_2_), 3.01 (t, *J* = 6.4 Hz, 2H, CH_2_), 3.21–3.30 (m, 4H, CH_2_), 3.30–3.37 (m, 2H, CH_2_), 3.61–3.72 (m, 4H, CH_2_), 4.48–4.59 (m, 4H, CH_2_), 4.54 (t, *J* = 6.2 Hz, 2H, CH_2_), 7.20–7.21 (m, 2H, aryl H), 7.24 (d, *J* = 8.0 Hz, 2H, aryl H), 7.67 (td, *J* = 7.6, 1.5 Hz, 2H, aryl H), 8.14 (d, *J* = 4 Hz, 2H, aryl H), 8.22 (s, 1H, aryl H); ^13^C-NMR (CDCl_3_) δ 17.8, 19.9, 21.2, 22.1,25.1, 25.6, 28.5, 29.3, 48.9, 51.5, 52.2, 53.8, 119.48 (q, *J*_CF_ = 320 Hz, CF_3_), 122.6, 123.8, 127.3, 137.6, 140.5, 148.4, 156.9; ESI-HRMS *m/z* [M]^+^ calcd for C_26_H_37_N_6_ 433.3080, found 433.3084.

### 3.7. Synthesis of 6-Bromohexanoic Acid N-Hydroxysuccinimide Ester

To a round bottom flask with a stir bar, 6-bromohexanoic acid (1.0 g, 5.12 mmol), *N*-hydroxysuccinimide (0.89 g, 7.69 mmol), 4-dimethylaminopyridine (0.16 g, 1.02 mol), and CH_2_Cl_2_ (12 mL) was added. The solution of 1-(3-dimethylaminopropyl)-3-ethylcarbodiimide hydrochloride (1.18 g, 6.14 mmol) in dichloromethane (30 mL) added in small portions solution to the round bottle in ice bath, then the mixture was allowed to react at ambient temperature for 2 h. When the reaction was completed, washed sequentially with citric acid (10 wt %), sodium bicarbonate (10 wt %) and brine. The organic washings were dried over Na_2_SO_4_ and the solvent was removed under reduced pressure to afford 6-bromohexanoic acid N-hydroxysuccinimide ester (1.27 g, 85% yield) as a white solid.

### 3.8. Synthesis of N,N-Bis(2-(pyridin-2-yl)ethyl)hexan-1-amine *(**4**)*

To a solution of *n*-hexylamine (3.25 g, 3.21 mmol) in MeOH (2 mL) was added 2-vinylpyridine (2 equiv.) and acetic acid (3 equiv.). The resulting solution was allowed to reflux at 65 °C for 72 h. After the reaction was completed, the solvent and 2-vinylpyridine were removed under reduced pressure. The mixture product was adjusted to pH 10 using concentrated aqueous NaOH (1 M), and the resulting mixture was extracted with ethyl acetate. The resulting organic phase was dried over Na_2_SO_4_. After filtration and solvent evaporation under reduced pressure, the crude product mixture was purified by flash column chromatography to afford affinity ligand *N*,*N*-bis(2-(pyridin-2-yl)ethyl)hexan-1-amine (**4**, 0.7534 g, 75% yield) as a brown oil. ^1^H-NMR (CDCl_3_) δ 0.87 (t, CH_3_, 3H, *J* = 7.0 Hz), 1.12–1.35 (m, CH_2_, 6H), 1.35–1.49 (m, CH_2_, 2H), 2.54 (t, CH_2_, 2H, *J* = 7.6 Hz), 2.93 (bs, CH_2_, 8H), 7.01–7.15 (m, aryl H, 4H), 7.56 (td, aryl H, 2H, *J* = 7.2, 1.2 Hz), 8.52 (d, aryl H, 2H, *J* = 3.2 Hz); ^13^C-NMR (CDCl_3_) δ 14.0, 22.6, 27.1, 27.2, 31.8, 35.9, 54.0, 54.0, 121.0, 123.3, 136.1, 149.1, 160.8; ESI-HRMS *m/z* [M + H]^+^ calcd for C_20_H_30_N_3_ 312.2440, found 312.2444.

### 3.9. Binding Stoichiometry Measurement of Cu(OTf)_2_ with Affinity Ligand ***4***

Stoichiometric titrations were performed with 200 mM affinity ligand **4** (10 µL) in DMSO/H_2_O solution (5:1, *v*/*v*). The solution of affinity ligand **4** was titrated with an aqueous solution (10 µL) containing copper triflate (0–400 mM) in DMSO/H_2_O solution (5:1, *v*/*v*). Upon mixing, the resulting solutions were analyzed by UV-Vis spectrophotometry (NanoDrop 2000, Thermo Scientific, Waltham, MA, USA). The mixture solutions exhibit a new absorption band at λ = 680 nm. The absorbance (680 nm) of resulting solutions were plotted vs. the ratio of (Cu(II))/(affinity ligand **4**).

### 3.10. Mass Spectrometric Analysis of Cu(II)-Affinity Ligand ***4*** Complex

To a microtube with affinity ligand **4** (2.7 mg, 8.63 µmol) in methanol (1 mL) was added copper triflate (3.1 mg, 8.63 µmol). The mixture was allowed to react at ambient temperature for 10 min, followed by washing with ether three times. The solvent of the resulting mixture was removed under reduced pressure to afford the Cu complex as brown oil. This sample was analyzed by ESI-MS ESI-HRMS *m/z* [M + Na]^+^ calcd for C_22_H_29_N_3_O_6_F_6_Na_1_S_2_Cu_1_ 695.0596, found 695.0595.

### 3.11. Affinity Extraction of Cu(OAc)_2_ by ***AIL 3*** and Its Back Extraction

To a microtube containing **AIL 3** (250 mM) in [e-4C-tr][NTf_2_] ionic liquid **2** (25 µL) was added copper acetate (100 mM, 25 µL). The two phases were vigorously shaken to extract metal ion. The mixture was then centrifuged to induce phase separation. The upper layer was measured to determine the concentration of copper acetate remained in aqueous layer by UV-vis spectrophotometry (NanoDrop 2000). The upper aqueous layer was removed and 1 M HCl solution (25 µL) was added to the tube for back extraction. The mixture solution was vigorously shaken and then centrifuged to induce phase separation. The upper layer was then measured to determine the concentration of copper chloride in the aqueous layer using the NanoDrop 2000 UV-Vis spectrophotometer.

### 3.12. Affinity Extraction of Cobalt Acetate and Nickel Acetate

To a microtube containing **AIL 3** (250 mM) in [e-4C-tr][NTf_2_] ionic liquid **2** (25 µL) was added cobalt acetate or nickel acetate (250 mM, 25 µL). The two phases were vigorously shaken to extract metal ion. The mixture was then centrifuged to induce phase separation. The upper layer was measured to determine the concentration of cobalt acetate or nickel acetate remained in aqueous layer by UV-vis spectrophotometry (NanoDrop 2000).

### 3.13. Regeneration of ***AIL 3***

To a microtube with Cu(II)-chelated **AIL 3** in ionic liquid **2** (25 µL) was added 1 M HCl aqueous solution (25 µL). This solution was vigorously shaken and then centrifuged to induce phase separation. The upper aqueous solution was removed and an aqueous solution containing 0.1 M Li_2_CO_3_ (100 µL) was added to wash the bottom ionic liquid layer five times continuously. After the final Li_2_CO_3_ aqueous solution was removed, the **AIL 3** in ionic liquid **2** was regenerated and ready for experiments.

### 3.14. Affinity Extraction of Dabsylated Pentapeptide by Cu(ll)-Chelated ***AIL 3*** and Back Extraction

To a microtube containing **AIL 3** (100 mM) in [b-4C-tr][NTf_2_] **1** ionic liquid (25 µL) was added copper acetate (50 mM) in water (25 µL). The two phase were vigorously shaken to extract metal ion, then the mixture was centrifuged to induce phase separation. After removing the upper aqueous layer, the dabsylated pentapeptide amide solution (1 mM) in 0.2 M NaOAc buffer (pH 4.4) containing 10% DMSO (25 µL) was added to microtube. After extraction and centrifuge, the upper layers were measured to determine the concentration of dabsylated pentapeptide in aqueous layer by UV-vis spectrophotometry. The upper aqueous solution was removed and the 1 M HCl solution (25 µL) was added to the microtube for back extraction. The solution was vigorously shaken and then centrifuged to induce phase separation. The upper HCl solution was turned red color indicating the peptide being successfully back-extracted.

## 4. Conclusions

We have developed a high yielding synthesis of bicyclic 1,2,3-triazolium ionic liquids that appear to fulfill practical requirement as solvents for chemical reactions. It is noted that this general synthesis established a common bicyclotriazole core so that derivatives of triazolium ionic liquids could be prepared, characterized and studied. Ionic liquids **1** and **2** were used to demonstrate their usefulness as ionic solvents for the synthesis of tryptanthrin and, likely, other natural and non-natural products. Lastly, an affinity ionic liquid **3** was synthesized and used to demonstrate its value in affinity extraction of both Cu(II) ion and His-containing peptides. Binding interaction study of **AIL 3**-Cu(II) complex with various biomolecules as well as experiments on reuse ability of our affinity system are actively being pursued and the result will be reported in due course.

## Figures and Tables

**Table 1:** Synthesis of tryptanthrin in ionic (**1**, **2**) and molecular (DMF, toluene) solvents ^a^.

Entry	Base	Solvent	Isolated Yield
1	DIEA	ionic liquid **1**	99%
2	Cs_2_CO_3_	ionic liquid **1**	81%
3	DIEA	ionic liquid **2**	98%
4	DIEA	DMF	64%
5	Cs_2_CO_3_	DMF	21%
6	--	ionic liquid **1**	19% ^b^
7	--	DMF	6% ^b^
8	DIEA	toluene	no reaction
9	Cs_2_CO_3_	toluene	no reaction

^a^ Experimental conditions: isatoic anhydride (0.1 mmol), isatin (0.1 mmol), base (0.1 mmol), solvent (50 μL), microwave at 100 °C (30 W) for 2 min. ^b^ condition: isatoic anhydride (0.1 mmol), isatin (0.1 mmol), solvent (50 μL), microwave at 180 °C (30 W) for 10 min.

## Supplementary Materials

Supplementary materials can be accessed at: http://www.mdpi.com/1420-3049/21/10/1355/s1.
