# Supplementary material for: Exploiting 1,2,3-Triazolium Ionic Liquids for Synthesis of Tryptanthrin and Chemoselective Extraction of Copper(II) Ions and Histidine-Containing Peptides
Source: Molecules. 2016 Oct 13;21(10):1355. doi: 10.3390/molecules21101355 (PMC6274210; doi:10.3390/molecules21101355)
Supplement: Supplementary file 1 [file molecules-21-01355-s001.pdf]

Supplementary Materials: Exploiting 1,2,3-Triazolium Ionic Liquids for Synthesis of Tryptanthrin and Chemoselective Extraction of Copper(II) Ions and Histidine-Containing Peptides

Hsin-Yi Li, Chien-Yuan Chen, Hui-Ting Cheng and Yen-Ho Chu

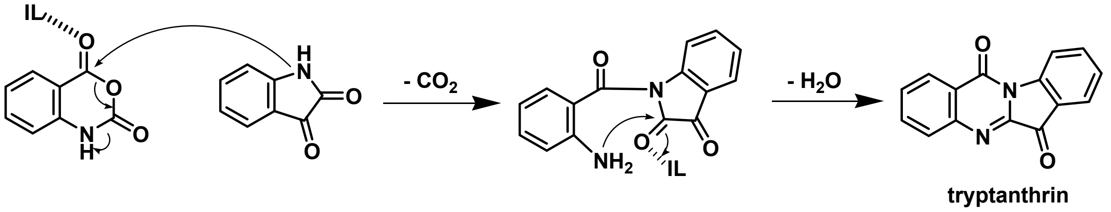

Figure S1. Possible mechanism for the synthesis of tryptanthrin in ionic liquid (IL) without the use of base. The weak Lewis acidity of ionic liquid may come from its bicyclic 1,2,3-triazolium cation.

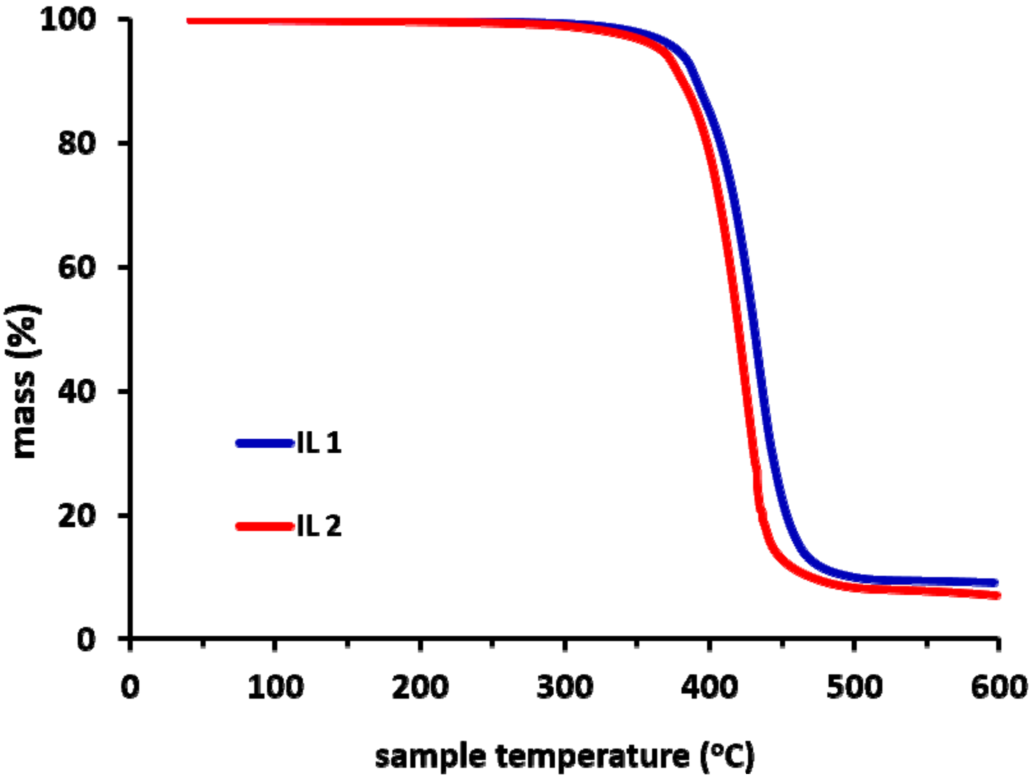

Figure S2. Thermogravimetric analysis (TGA) of bicyclic 1,2,3-triazolium ionic liquids **1** and **2** measured under nitrogen at a scanning speed of 20 °C·min^{-1}. T_{dcp} reflects the temperature at which a weight loss of 10% is observed: 391 °C and 382 °C for ionic liquid **1** and **2**, respectively.

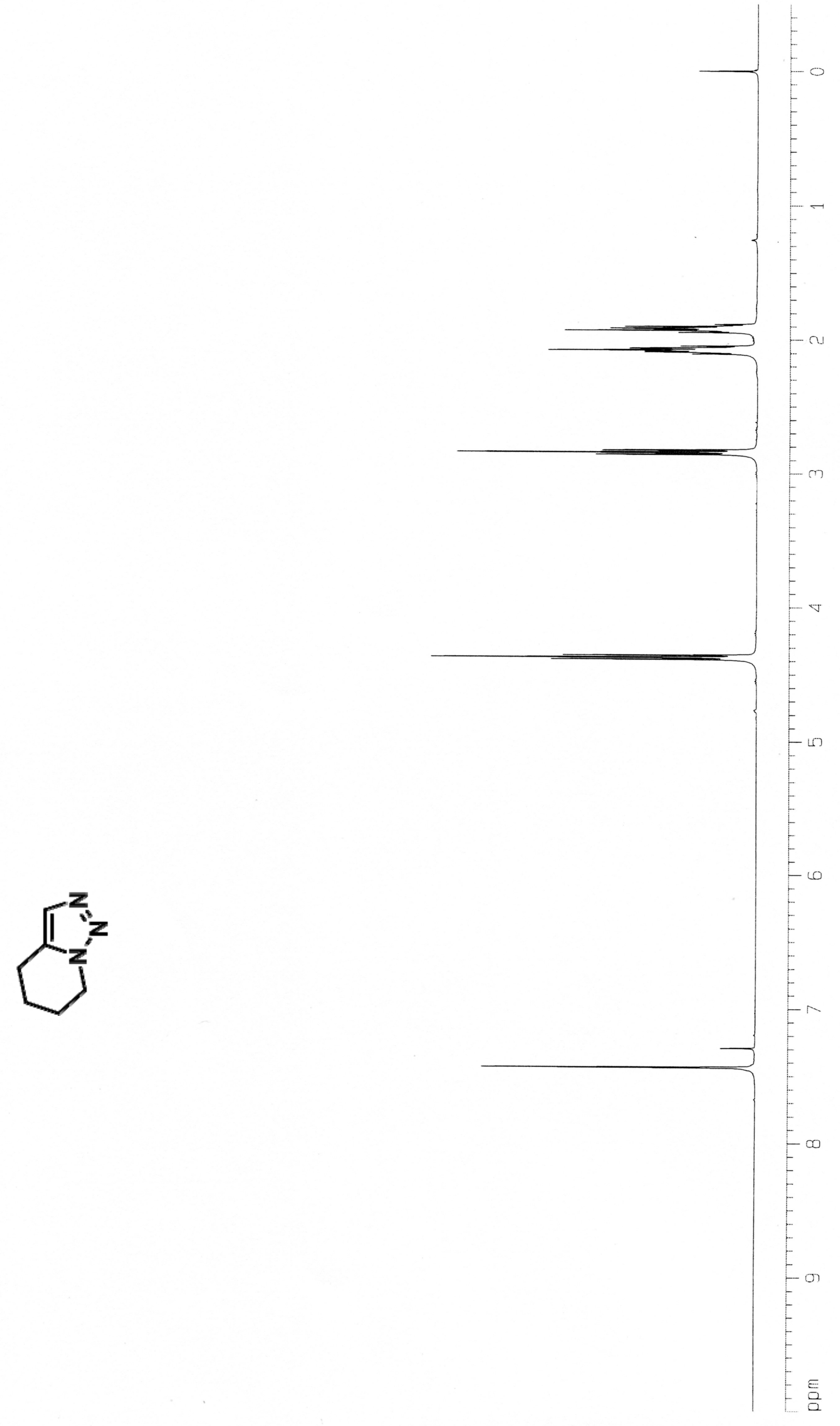

Figure S3. ^{1}H-NMR of 4,5,6,7-Tetrahydro[1,2,3]triazolo[1,5-*a*]pyridine.

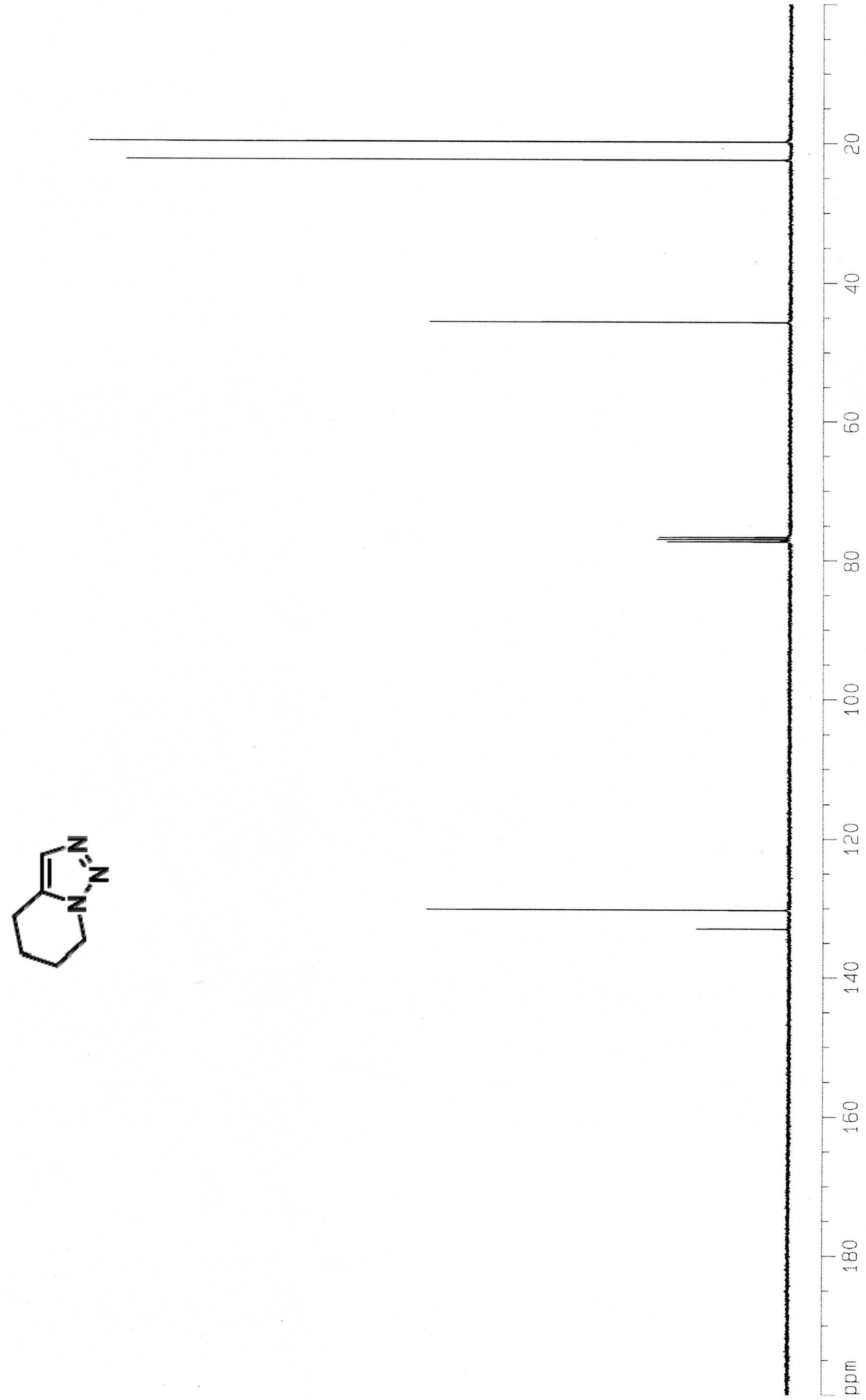

Figure S4. ^{13}C-NMR of 4,5,6,7-Tetrahydro[1,2,3]triazolo[1,5-*a*]pyridine.

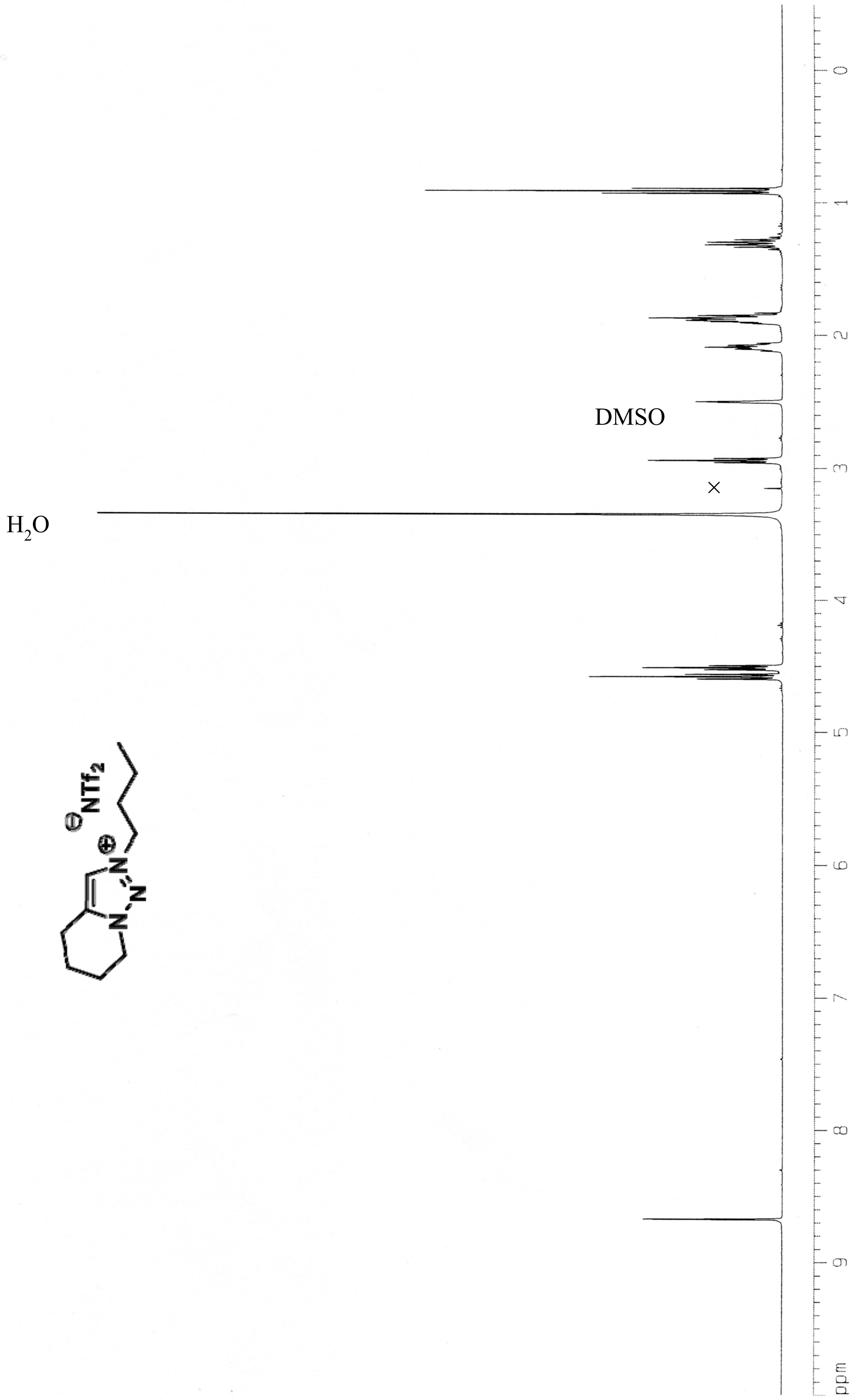

Figure S5. ^{1}H-NMR of Ionic Liquid [b-4C-tr][NTf2] (**1**).

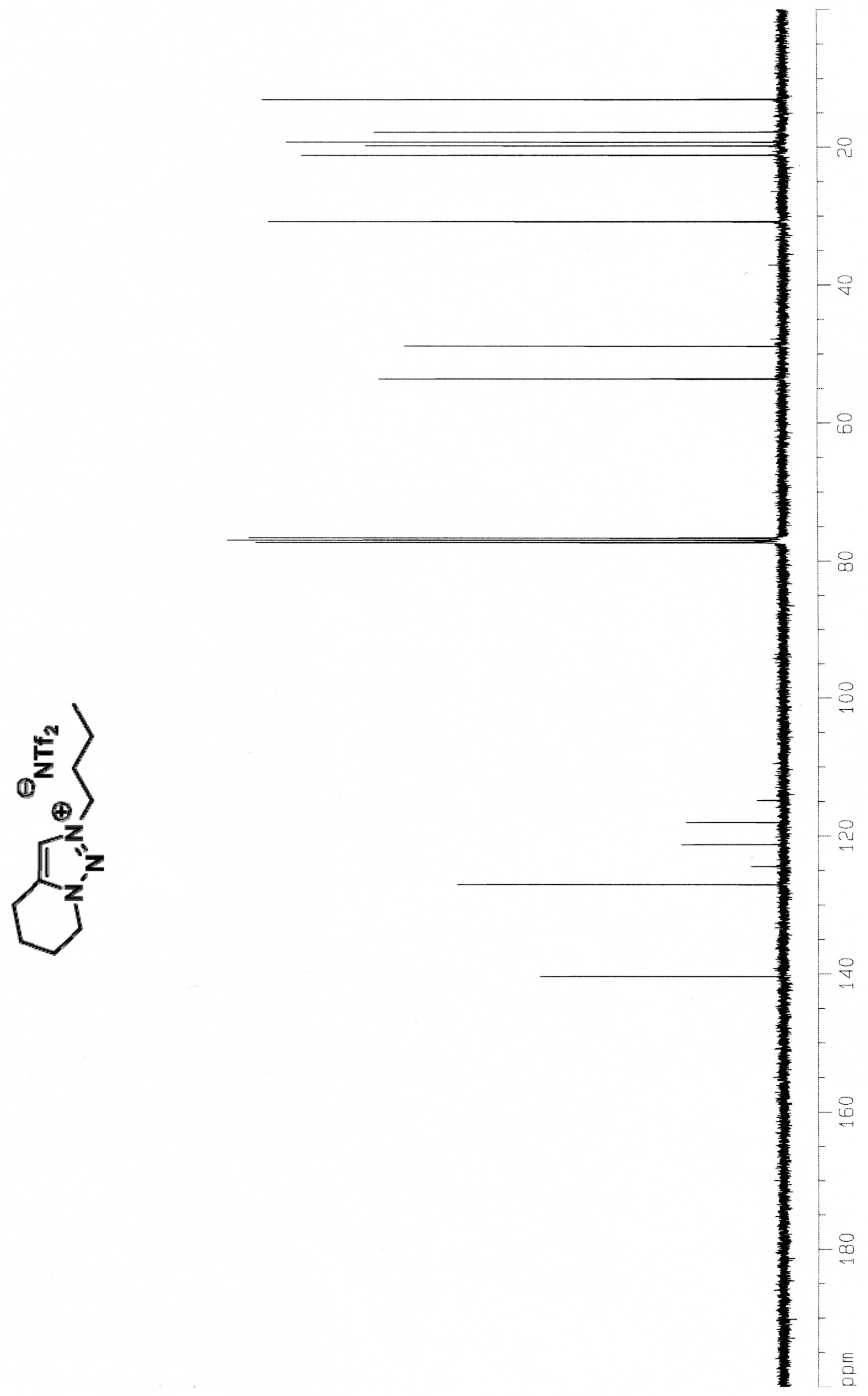

Figure S6. ^{13}C-NMR of Ionic Liquid [b-4C-tr][NTf_2] (**1**).

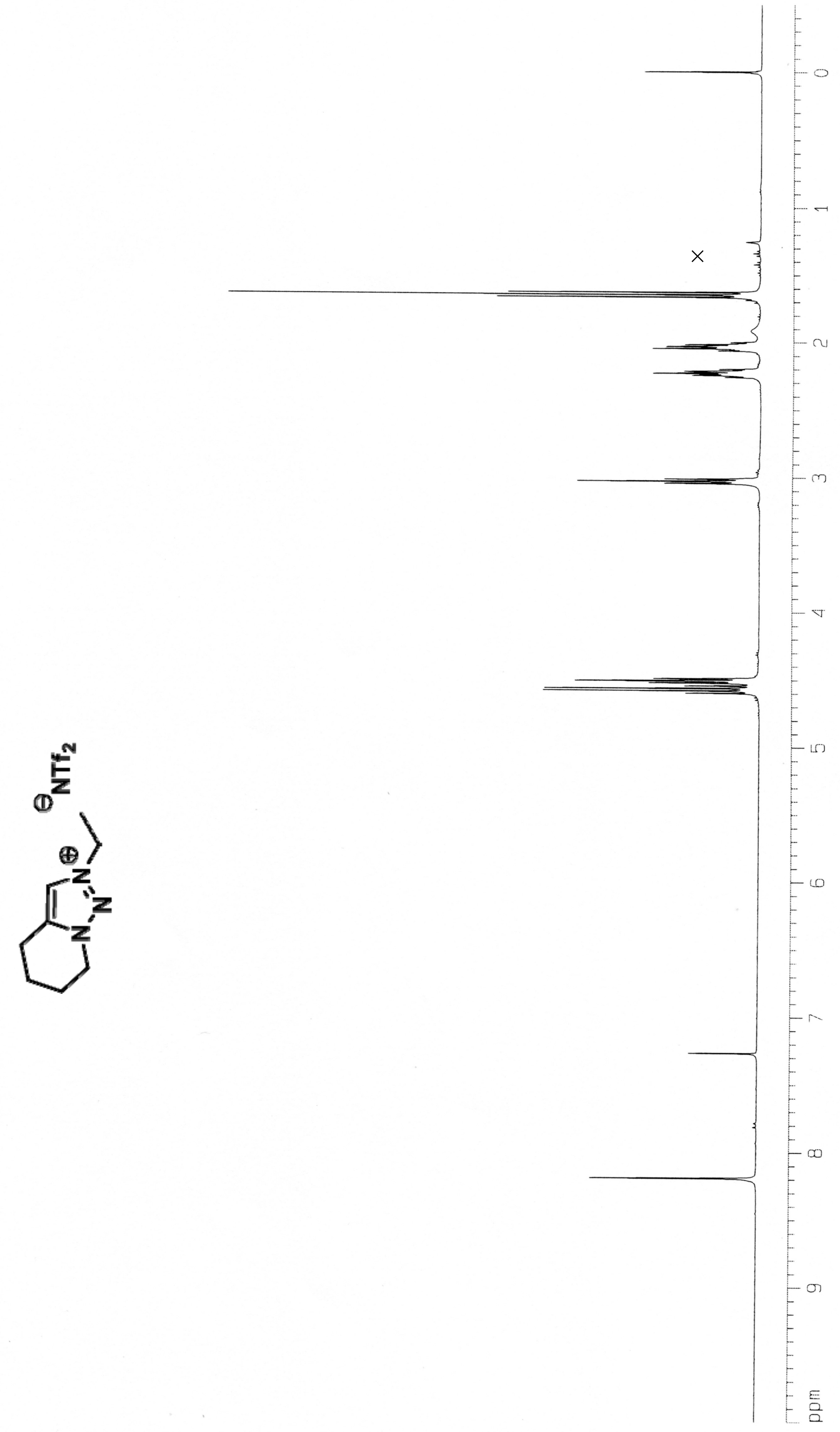

Figure S7. ^{1}H-NMR of Ionic Liquid [e-4C-tr][NTf_2] (**2**).

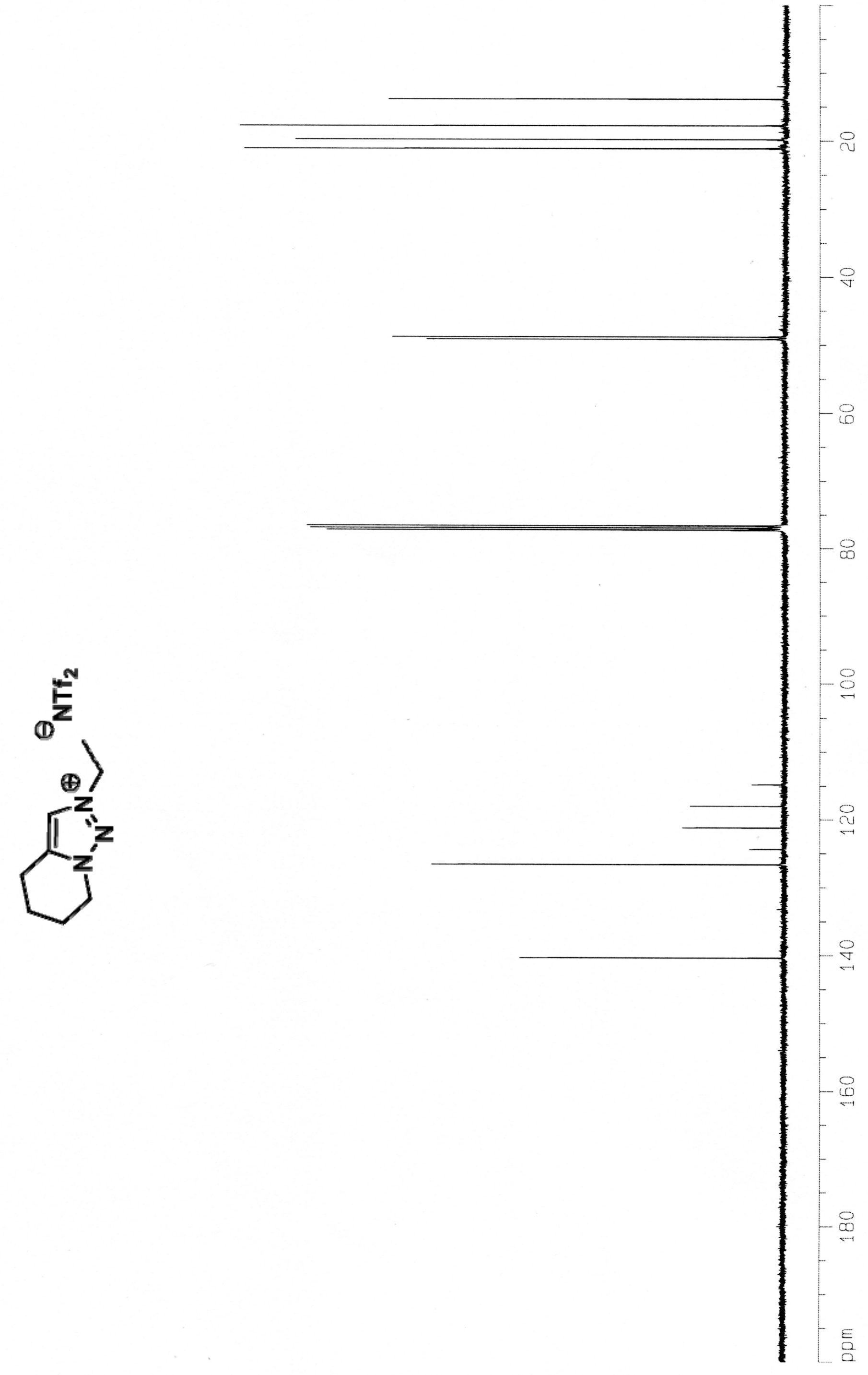

Figure S8. ^{13}C-NMR of Ionic Liquid [e-4C-tr][NTf2] (**2**).

2

[Elemental Composition] Page: 1
Data : 14Aug19_E02-001 Date : 19-Aug-2014 16:27
Sample: 2
Note : 70eV
Inlet : Direct Ion Mode : EI+
RT : 1.22 min Scan#: 12
Elements : C 400/0, H 800/0, N 3/3
Mass Tolerance : 10mmu
Unsaturation (U.S.) : -0.5 - 10000.0

Observed m/z	Int%	Err[ppm / mmu]	U.S.	Composition
152.1183	100.0	-3.0 / -0.5	3.5	C 8 H 14 N 3 = 152.1188

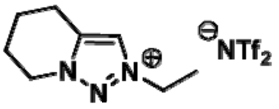

[Mass Spectrum]
Data : 14Aug19_E02-001 Date : 19-Aug-2014 16:27
Sample: 2
Note : 70eV
Inlet : Direct Ion Mode : EI+
Spectrum Type : Normal Ion [EF-Linear]
RT : 1.22 min Scan# : 12
BP : m/z 152.1183 Int. : 31.06
Output m/z range : 100.0000 to 200.0000 Cut Level : 0.00 %

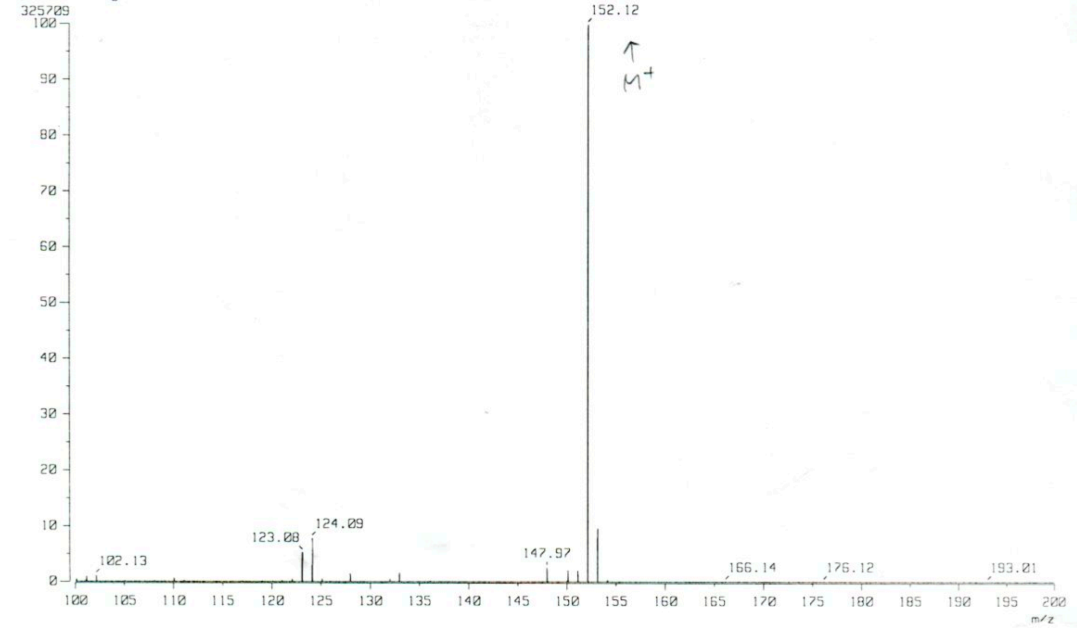

Figure S9. ESI-HRMS spectrum of ionic liquid [e-4C-tr][NTf2] (**2**).

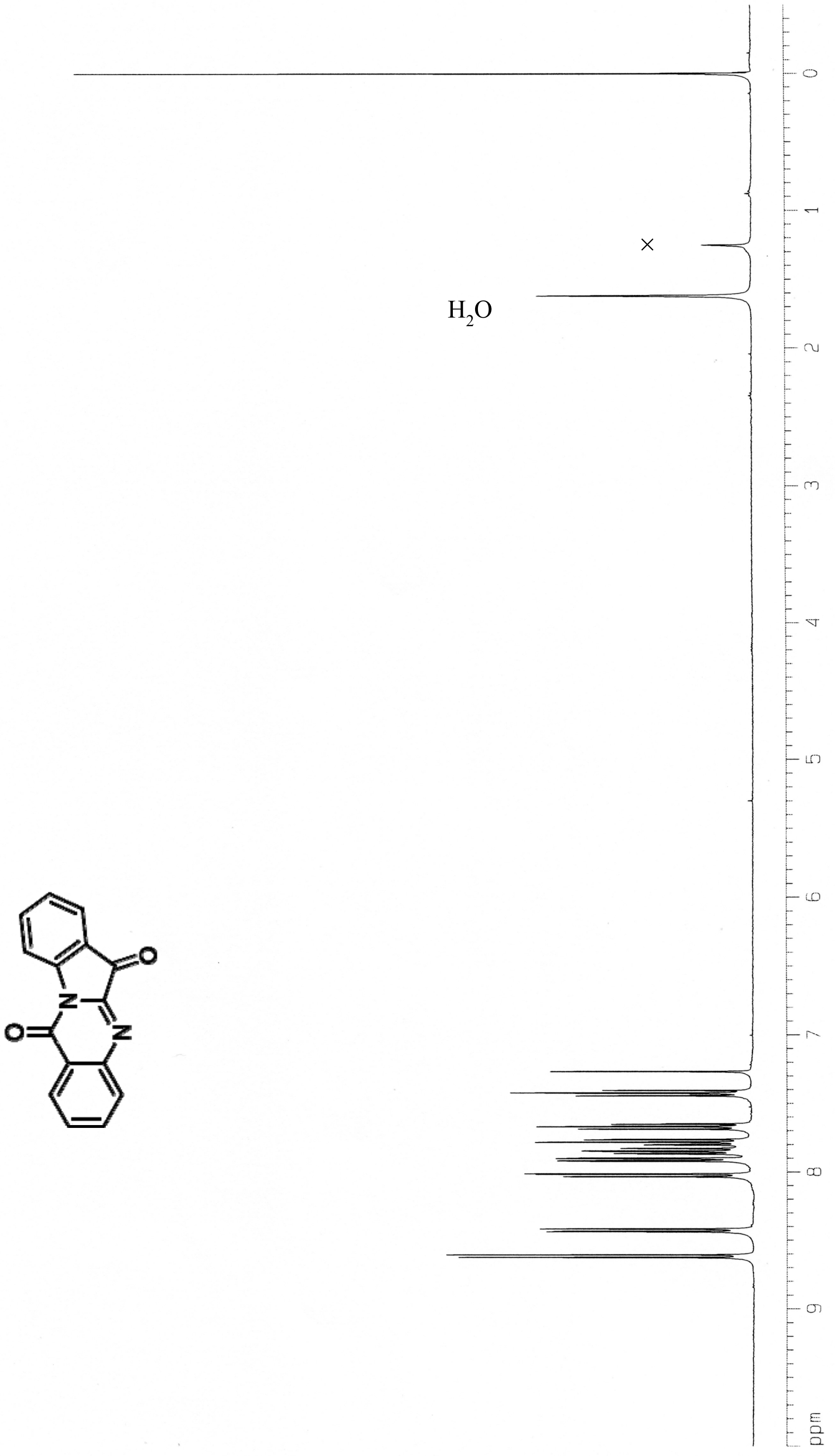

Figure S10. ^{1}H-NMR of tryptanthrin.

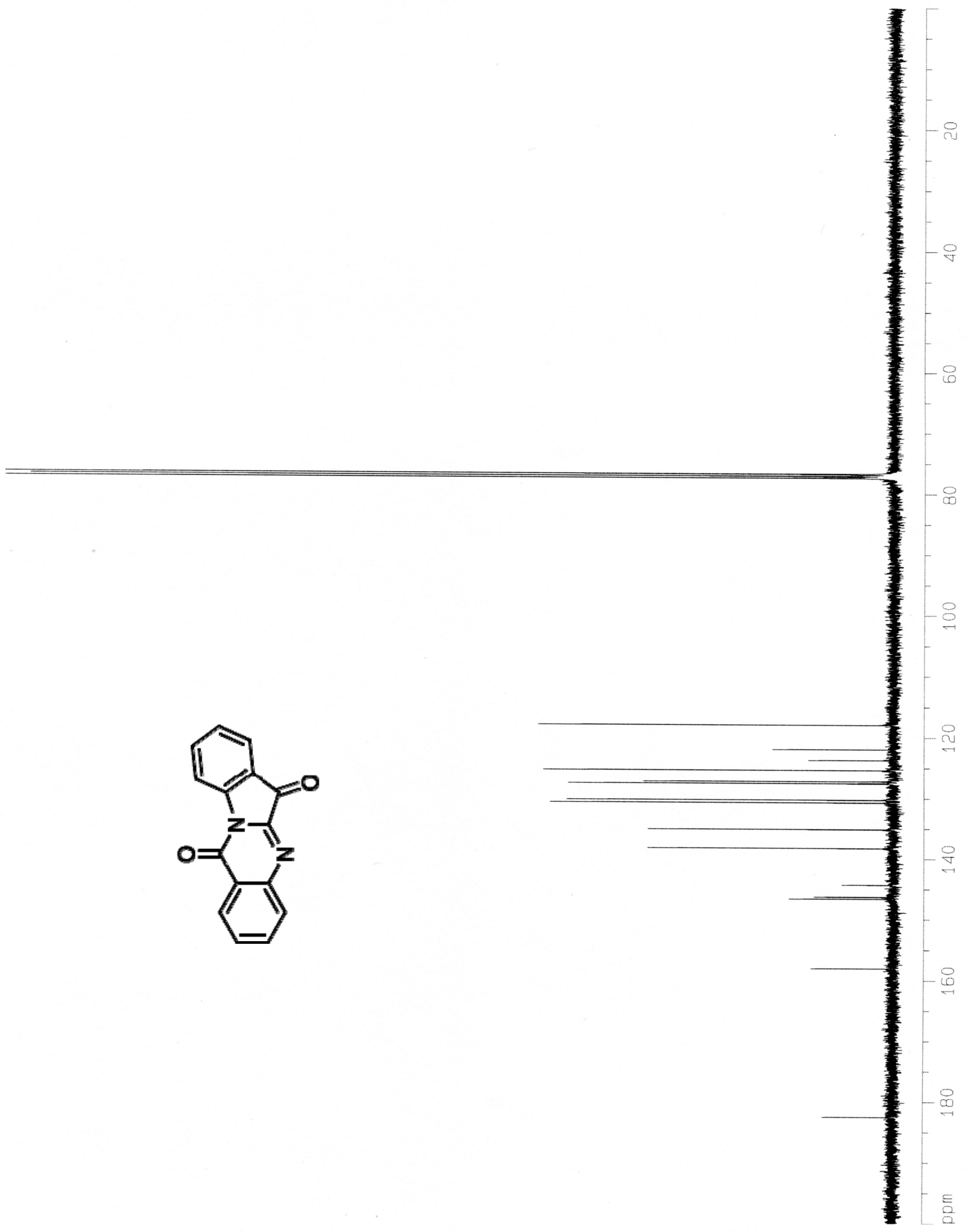

Figure S11. ^{13}C-NMR of tryptanthrin.

1

[Elemental Composition] Page: 1
Data : 14Aug19_E03-001 Date : 19-Aug-2014 16:35
Sample: 1
Note : 70eV
Inlet : Direct Ion Mode : EI+
RT : 0.15 min Scan#: 3
Elements : C 400/0, H 800/0, O 2/2, N 2/2
Mass Tolerance : 10mmu
Unsaturation (U.S.) : -0.5 - 10000.0

Observed m/z	Int%	Err[ppm / mmu]	U.S.	Composition
248.0586	100.0	+0.0 / +0.0	13.0	C 15 H 8 O 2 N 2 = 248.0586

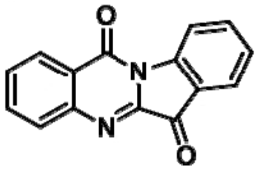

[Mass Spectrum]
Data : 14Aug19_E03-001 Date : 19-Aug-2014 16:35
Sample: 1
Note : 70eV
Inlet : Direct Ion Mode : EI+
Spectrum Type : Normal Ion [EF-Linear]
RT : 0.15 min Scan# : 3
BP : m/z 248.0586 Int. : 12.81
Output m/z range : 220.0000 to 300.0000 Cut Level : 0.00 %

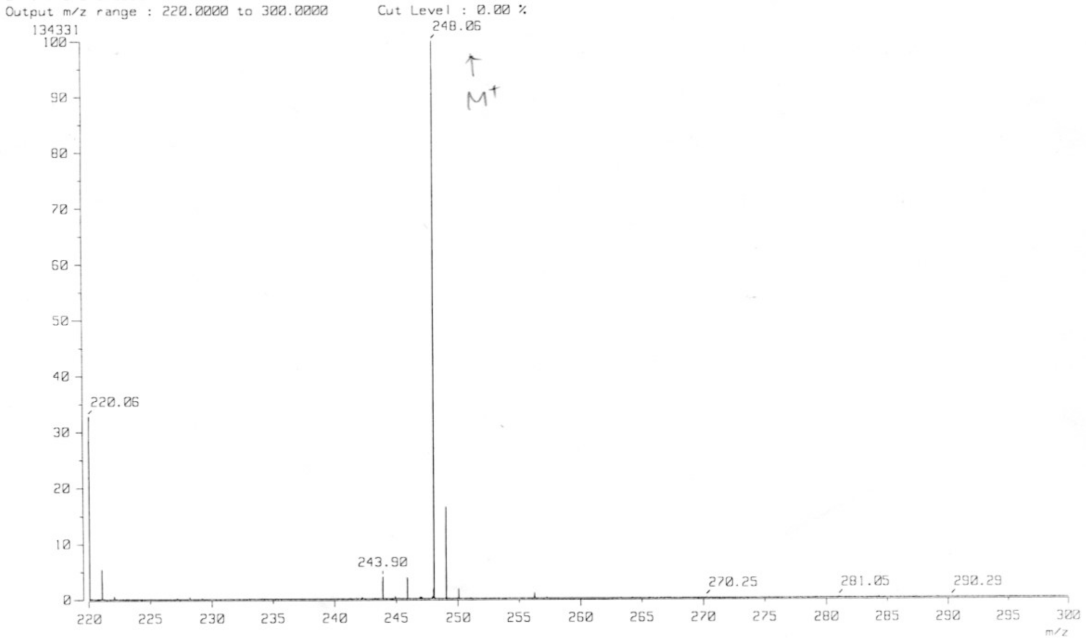

Figure S12. ESI-HRMS spectrum of tryptanthrin.

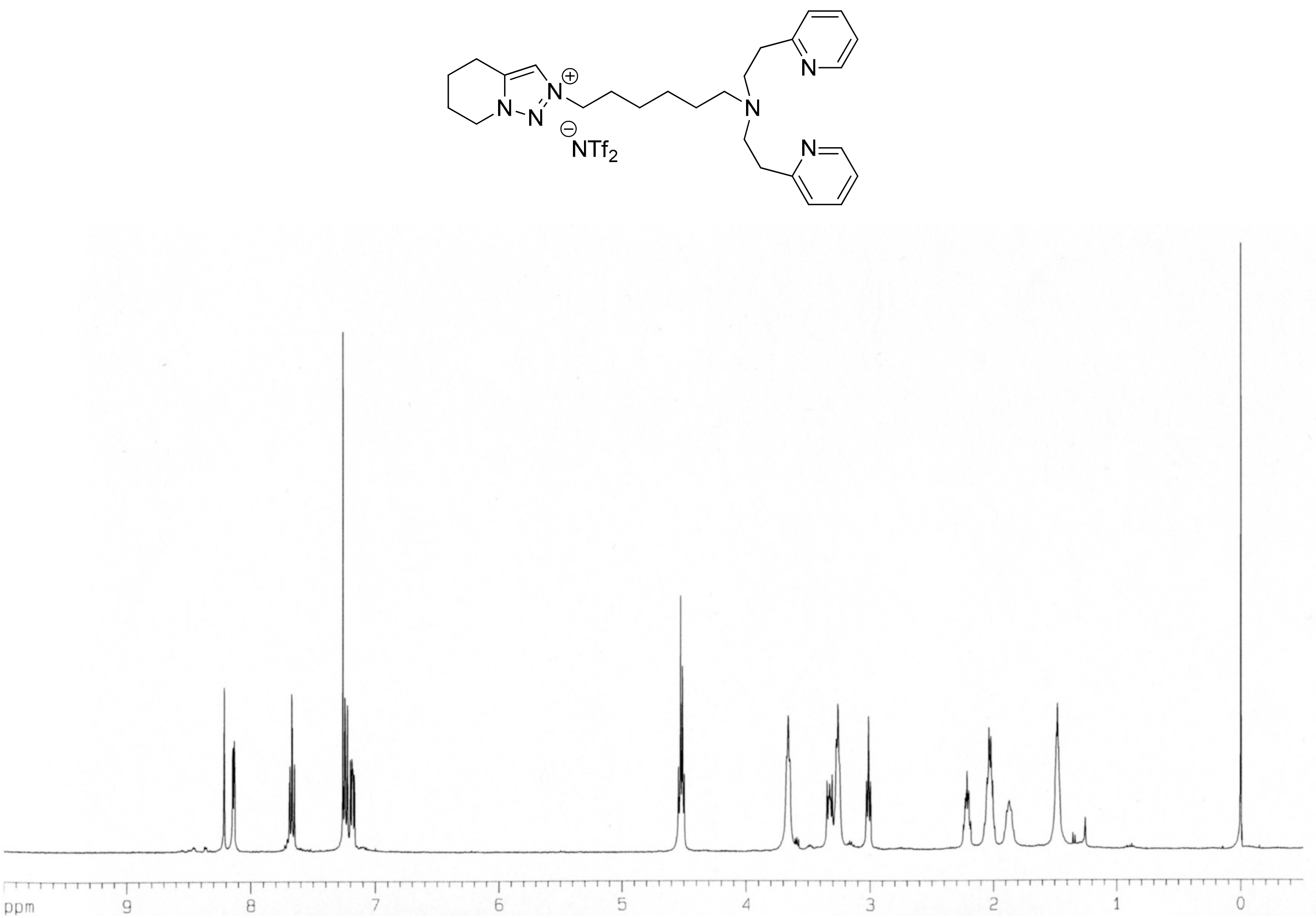

Figure S13. ^{1}H-NMR of Affinity Ionic Liquid **3**.

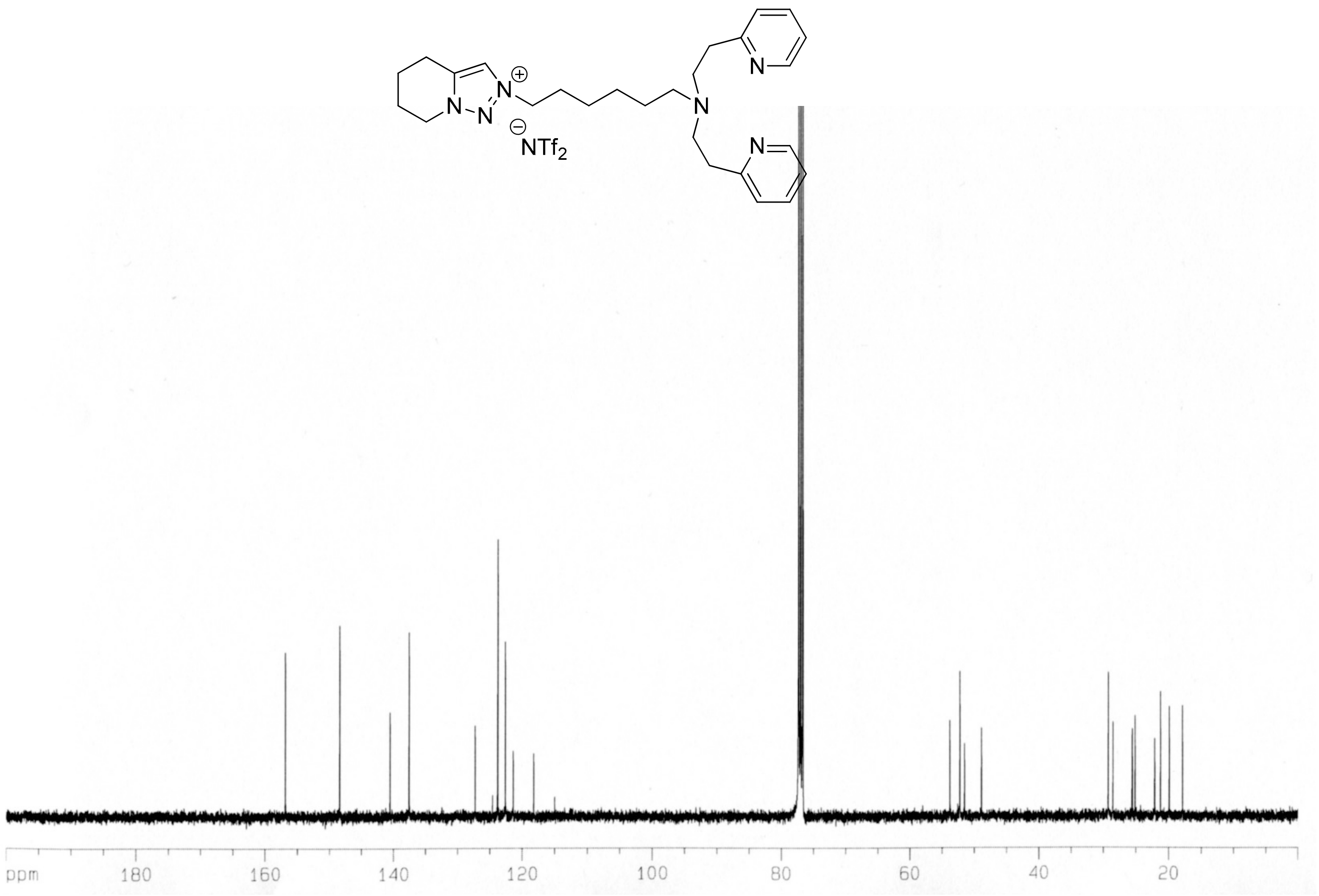

Figure S14. ^{13}C-NMR of Affinity Ionic Liquid **3.**

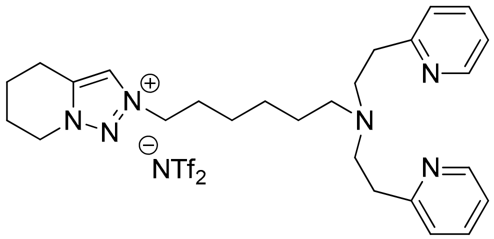

Elemental Composition Report **Page 1**

Single Mass Analysis
Tolerance = 50.0 PPM / DBE: min = -1000.0, max = 1000.0
Element prediction: Off
Number of isotope peaks used for i-FIT = 2

Monoisotopic Mass, Even Electron Ions
30 formula(e) evaluated with 1 results within limits (all results (up to 1000) for each mass)
Elements Used:
C: 0-1000 H: 0-1000 N: 6-6

1125-5 KE267 02-Dec-2015
16:23:31
1202_1125-5_2 3 (0.268) 1: TOF MS ES+
1.03e+005

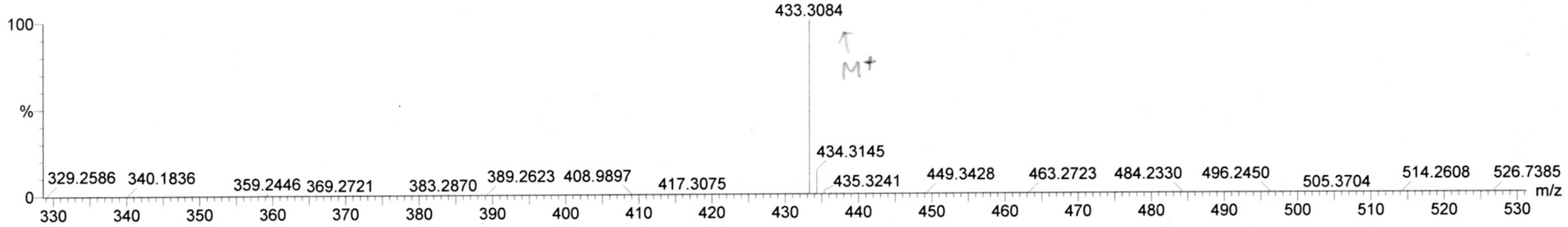

Minimum:				-1000.0			
Maximum:		5.0	50.0	1000.0			
Mass	Calc. Mass	mDa	PPM	DBE	i-FIT	i-FIT (Norm)	Formula
433.3084	433.3080	0.4	0.9	11.5	53.8	0.0	C26 H37 N6

Figure S15. ESI-HRMS spectrum of Affinity Ionic Liquid **3**.

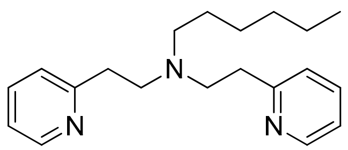

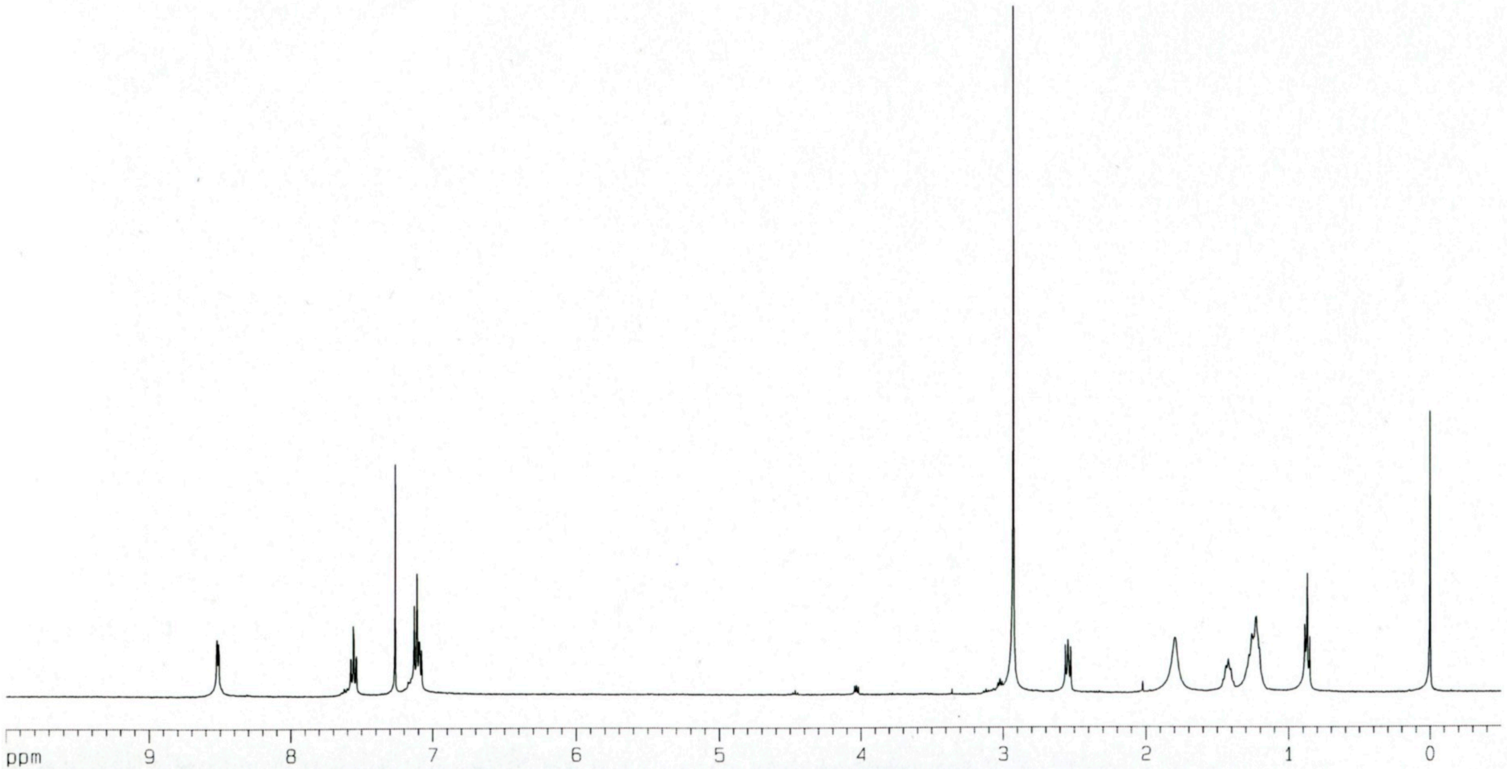

Figure S16. ^{1}H-NMR of *N*,*N*-bis(2-(pyridin-2-yl)ethyl)hexan-1-amine (**4**).

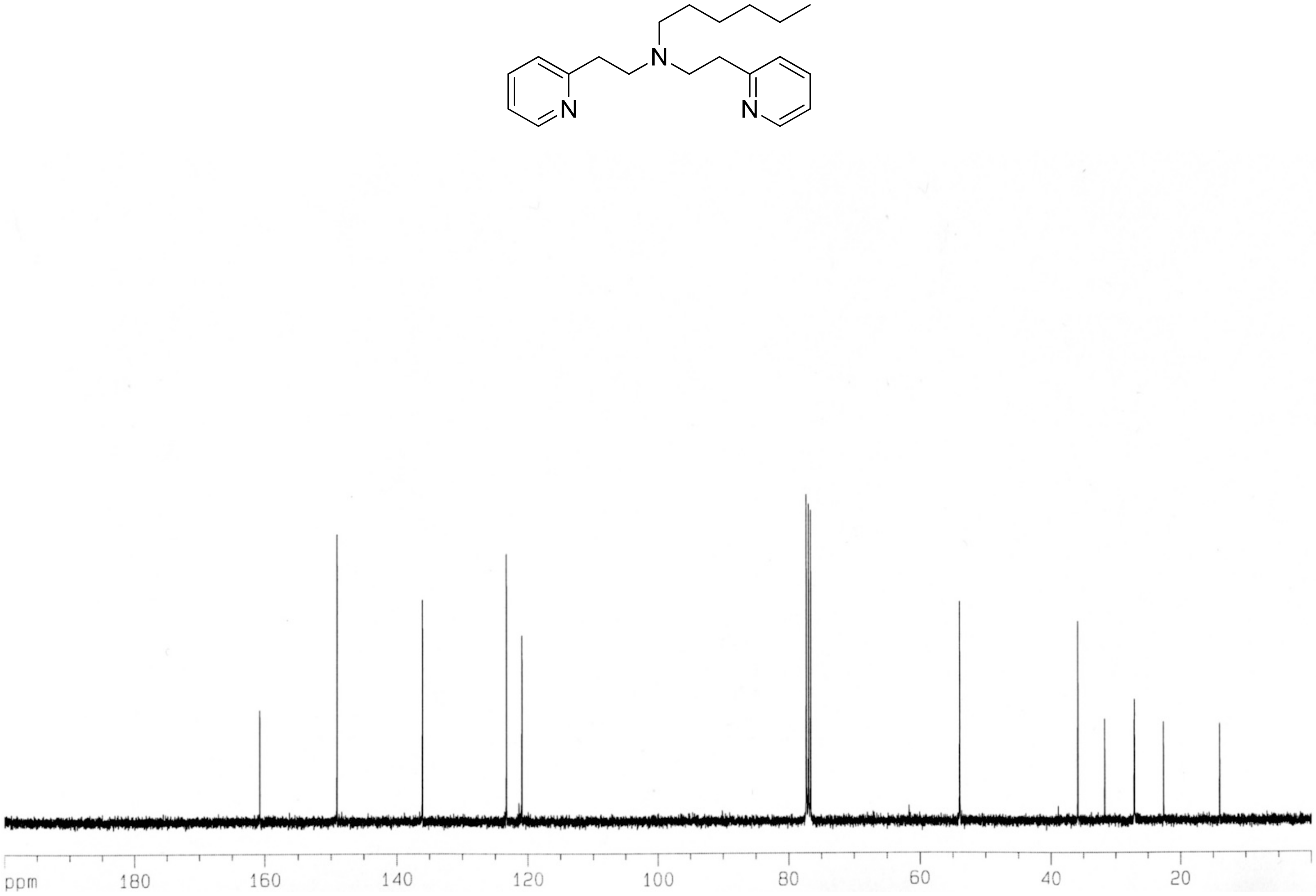

Figure S17. ^{13}C-NMR of *N*,*N*-bis(2-(pyridin-2-yl)ethyl)hexan-1-amine (**4**).

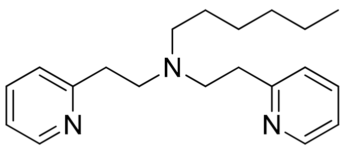

Elemental Composition Report **Page 1**

Single Mass Analysis

Tolerance = 70.0 PPM / DBE: min = -1000.0, max = 1000.0
Element prediction: Off
Number of isotope peaks used for i-FIT = 2

Monoisotopic Mass, Even Electron Ions
23 formula(e) evaluated with 1 results within limits (all results (up to 1000) for each mass)
Elements Used:
C: 0-1000 H: 0-1000 N: 3-3

1 KE267 02-Feb-2016 16:30:51

0202_1 3 (0.241) 1: TOF MS ES+ 7.84e+004

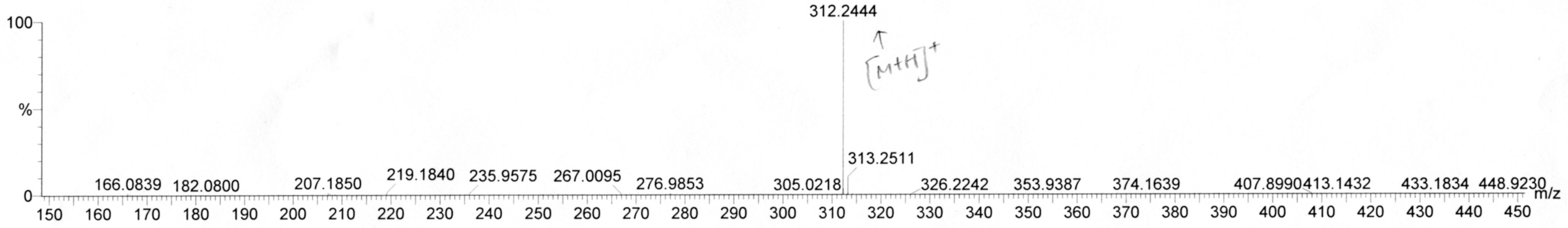

Minimum:				-1000.0			
Maximum:		5.0	70.0	1000.0			
Mass	Calc. Mass	mDa	PPM	DBE	i-FIT	i-FIT (Norm)	Formula
312.2444	312.2440	0.4	1.3	7.5	73.2	0.0	C20 H30 N3

Figure S18. ESI-HRMS spectrum of *N*,*N*-bis(2-(pyridin-2-yl)ethyl)hexan-1-amine (**4**).

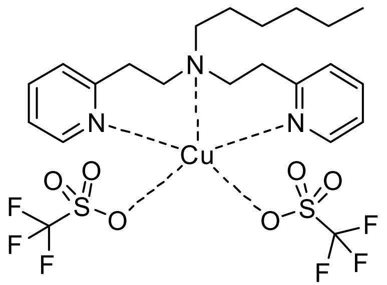

Elemental Composition Report **Page 1**

Single Mass Analysis
Tolerance = 7.0 PPM / DBE: min = -1000.0, max = 1000.0
Element prediction: Off
Number of isotope peaks used for i-FIT = 2

Monoisotopic Mass, Even Electron Ions
25 formula(e) evaluated with 1 results within limits (all results (up to 1000) for each mass)
Elements Used:
C: 0-1000 H: 0-1000 N: 3-3 O: 6-6 F: 6-6 Na: 1-1 S: 2-2 Cu: 1-1
2

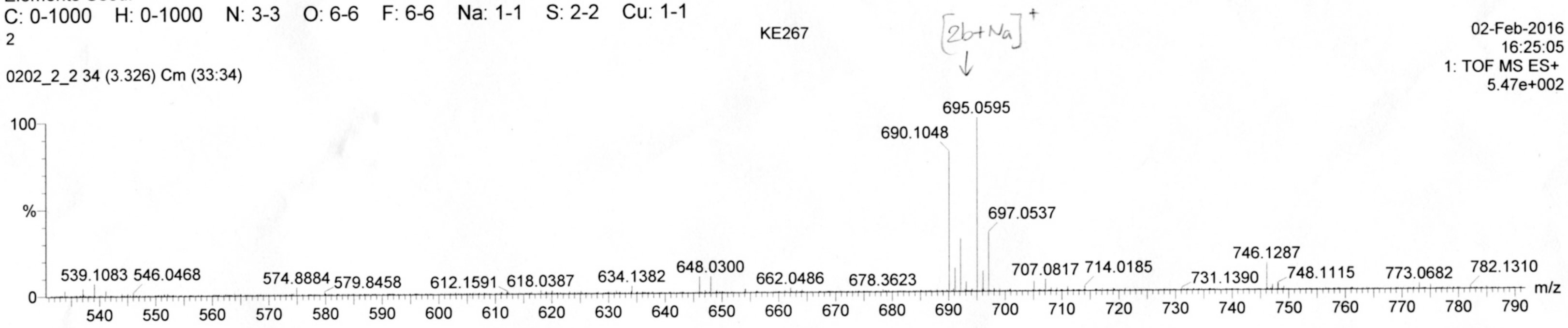

Minimum:				-1000.0			
Maximum:		5.0	7.0	1000.0			
Mass	Calc. Mass	mDa	PPM	DBE	i-FIT	i-FIT (Norm)	Formula
695.0595	695.0596	-0.1	-0.1	6.5	30.6	0.0	C22 H29 N3 O6 F6 Na S2 Cu

Figure S19. ESI-HRMS spectrum of Cu(II)-affinity ligand **4** Complex.
